# Dermal fibroblasts respond to IL-4 and IL-13 and promote T cell recruitment in atopic dermatitis

**DOI:** 10.1172/JCI196108

**Published:** 2026-01-22

**Authors:** Tomofumi Numata, Michael Shia, Yoshiyuki Nakamura, Fengwu Li, Hung Chan, Teruaki Nakatsuji, Kellen J. Cavagnero, Jared Simmons, Henry Li, Aaroh Anand Joshi, Marta Palomo-Irigoyen, Richard L. Gallo

**Affiliations:** Department of Dermatology, UCSD, La Jolla, California, USA.

**Keywords:** Dermatology, Immunology, Adaptive immunity, Allergy, Cytokines

## Abstract

Atopic dermatitis (AD) is a chronic inflammatory skin condition characterized by a type 2 immune response that is not fully understood. Single-cell RNA-seq of human AD skin and murine models of type 2 inflammation identified transcriptionally distinct fibroblast clusters, revealing IL-4Rα–dependent populations of immune-acting fibroblasts (IAFs). These unbiased findings prompted further investigation into the role of dermal fibroblasts during allergic inflammation. These studies demonstrated that, in an inflammatory environment including TNF-α, IL-1β, and IL-17A, the cytokines IL-4 and IL-13 stimulated both mouse and human fibroblasts to produce multiple chemokines, including CCL8, which activated CCR3 to attract T cells. In the skin, fibroblasts were the primary source of many of these chemokines, and targeted deletion of IL-4Rα in mouse fibroblasts reduced T cell infiltration in a mouse model of AD. Additionally, pharmacologic inhibition of CCR3, the receptor shared by many chemokines produced by fibroblasts, decreased T cell infiltration and skin inflammation in mouse models of AD. These findings demonstrate that dermal fibroblasts are more than passive structural cells; they actively participate in the type 2 immune response and contribute to AD by producing chemokines that increase inflammation. Targeting the functions of IAFs could offer an alternative therapeutic approach for AD.

## Abstract 2

Dermal fibroblasts respond to IL4 and IL13, produce Ccr3-binding chemokines, and drive T cell recruitment and inflammation in the skin.

## Graphical abstract

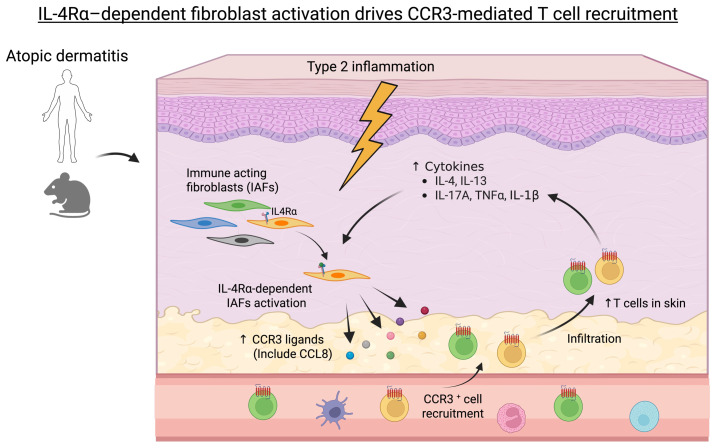

## Introduction

Atopic dermatitis (AD) is a chronic skin disease characterized by impaired epidermal barrier function, type 2 immune polarization, microbial dysbiosis, pruritus, and inflammation (1). Central to the pathogenesis of AD are the cytokines IL-4 and IL-13, which promote IgE production, eosinophil recruitment, and suppression of antimicrobial peptides (AMPs) (2–4). These type 2 cytokines orchestrate the activation and recruitment of a wide array of immune and nonimmune cells, including T cells, DCs, macrophages, type 2 innate lymphoid cells (ILC2s), mast cells, and keratinocytes (4–6). The critical role of these ILs in human AD is supported by clinical experience with drugs that inhibit the IL-4 receptor α (IL-4Rα) and IL-13 (7–11).

While keratinocytes have been extensively studied for their role in initiating and amplifying type 2 inflammation in AD, relatively little attention has been paid to the role of other cells that form the skin. Fibroblasts, the most abundant cell type in the dermis, have long been considered passive structural components or mesenchymal stem cells (12). However, emerging evidence indicates that fibroblasts are actively involved in immune modulation across various inflammatory contexts, including psoriasis, wound healing, bacterial infection, and inflammatory memory in chronic skin diseases (13–15). They can sense pathogen-associated or damage-associated molecular patterns via pattern recognition receptors (PRRs) and respond to cytokines by producing chemokines and other inflammatory mediators (16, 17). While such immune functions of fibroblasts are increasingly appreciated, their specific contributions to the immune landscape of AD remain poorly characterized (18).

Studies have demonstrated that *Staphylococcus aureus* (hereafter termed SA), a common colonizer of AD skin, can penetrate the compromised epidermal barrier and populate the dermis (19, 20). This suggests that dermal fibroblasts may be directly exposed to microbial and cytokine cues in situ, potentially contributing to local immune responses. Although biologic therapies targeting IL-4/IL-13 signaling have significantly improved AD treatment outcomes, their effects vary among patients and may lead to systemic side effects (4, 21). Moreover, emerging therapeutic strategies such as IL-1 receptor (IL-1R)–associated kinase 4 (IRAK4) inhibition are being investigated for broader suppression of inflammatory signaling, including fibroblast and keratinocyte responses (22). Therefore, gaining deeper insight into stromal-immune interactions — particularly the role of fibroblasts — may reveal therapeutic targets with improved tissue specificity and safety profiles.

Here, we hypothesized that dermal fibroblasts respond directly to type 2 cytokines by upregulating specific chemokines, thereby mediating inflammation in AD. To test this hypothesis, we performed single-cell RNA-seq (scRNA-seq) of human and mouse AD skin, followed by in vitro and in vivo functional experiments to evaluate the contribution of fibroblast-derived chemokines to allergic skin inflammation.

## Results

### Type 2 inflammation enhances chemokine expression by dermal fibroblasts.

**Figure 1:**
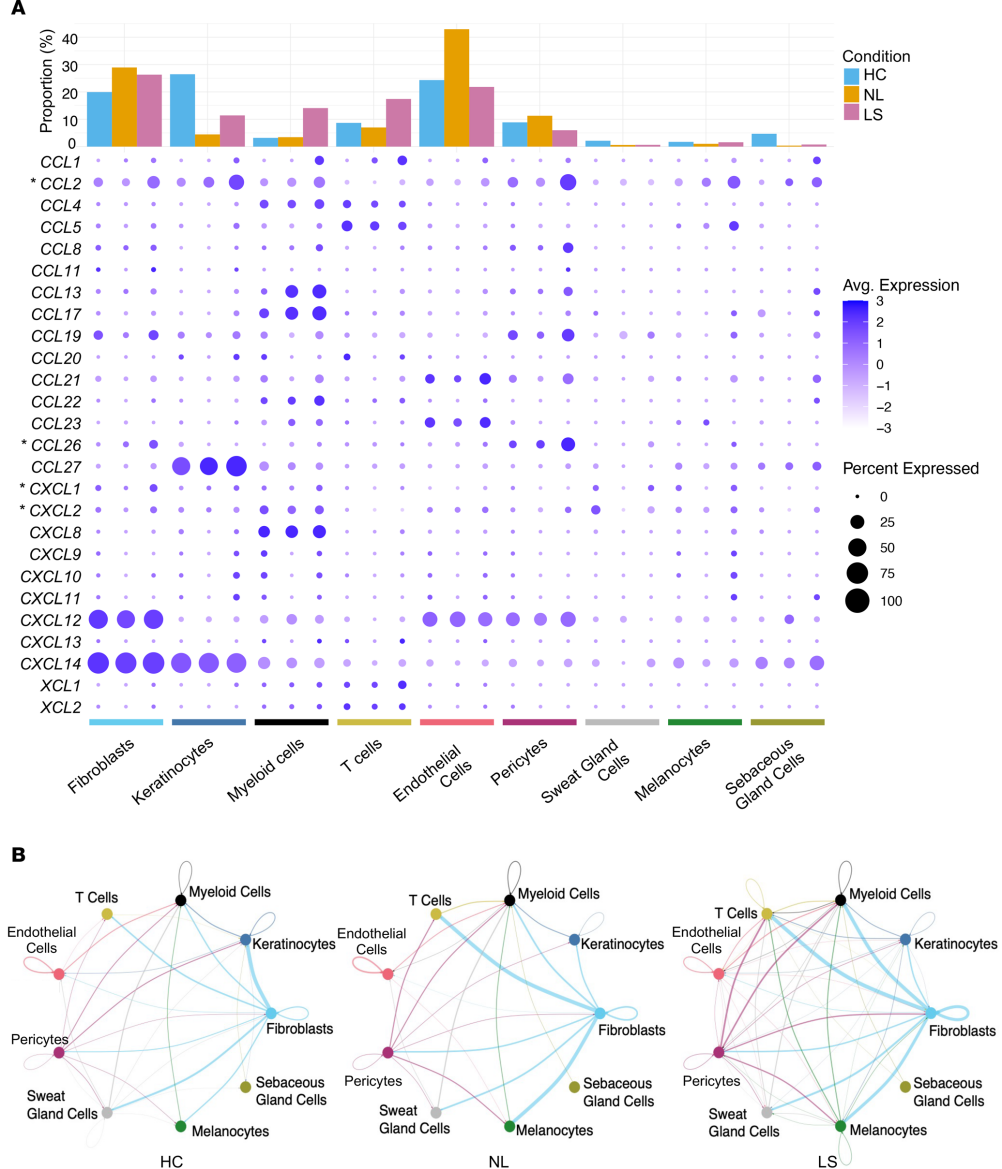
Transcriptional analysis of skin in AD shows increased chemokine gene expression by fibroblasts. (**A**) Bar plot for proportion of each cell type identified in HC (*n* = 8), NL (*n* = 5), and LS (*n* = 4) samples and dot plots showing chemokine expression. (**B**) Intercellular communication analysis between cell types in HC, NL, and LS samples using CellChat.

Fibroblasts have been hypothesized to play a pivotal role in coordinating immune responses (15, 23, 24), but their function in AD is unknown. To investigate the cell-specific changes in gene expression by dermal fibroblasts in AD, we performed unbiased network analysis of scRNA-seq data previously obtained from patients with AD (Gene Expression Omnibus [GEO] GSE147424) (25). On the basis of conventional marker gene expression, major cell lineages were identified, and cells were clustered as fibroblasts, keratinocytes, myeloid cells, T cells, endothelial cells, pericytes, sweat gland cells, melanocytes, and sebaceous gland cells (Supplemental Figure 1, A and B; supplemental material available online with this article; https://doi.org/10.1172/JCI196108DS1). As expected, the frequencies of myeloid cells and T cells increased in the skin of individuals with AD (Figure 1A). A dot plot visualizing changes in gene expression across all cell types and conditions revealed increased chemokine expression by several cell types in lesional skin from individuals with AD, with distinct patterns observed between fibroblasts, keratinocytes, myeloid cells, T cells, and pericytes. Further analysis of these data identified a total of 16 fibroblast clusters that could be distinguished between those seen in healthy human control skin samples compared with nonlesional and lesional AD skin (Supplemental Figure 1C). Eight functional characteristics were identified in the 16 fibroblast clusters, with several fibroblast clusters showing significant expression of chemokines (Supplemental Figure 1, D–U). Intercellular network analysis using CellChat further found communication from fibroblasts to myeloid cells and T cells that increased in AD (Figure 1B). These observations bring into question current models of AD pathogenesis in which keratinocytes and DCs are thought to be primarily responsible for recruiting immune cells following epidermal barrier disruption (26) and support the hypothesis that fibroblasts also participate in driving inflammation in AD.

**Figure 2:**
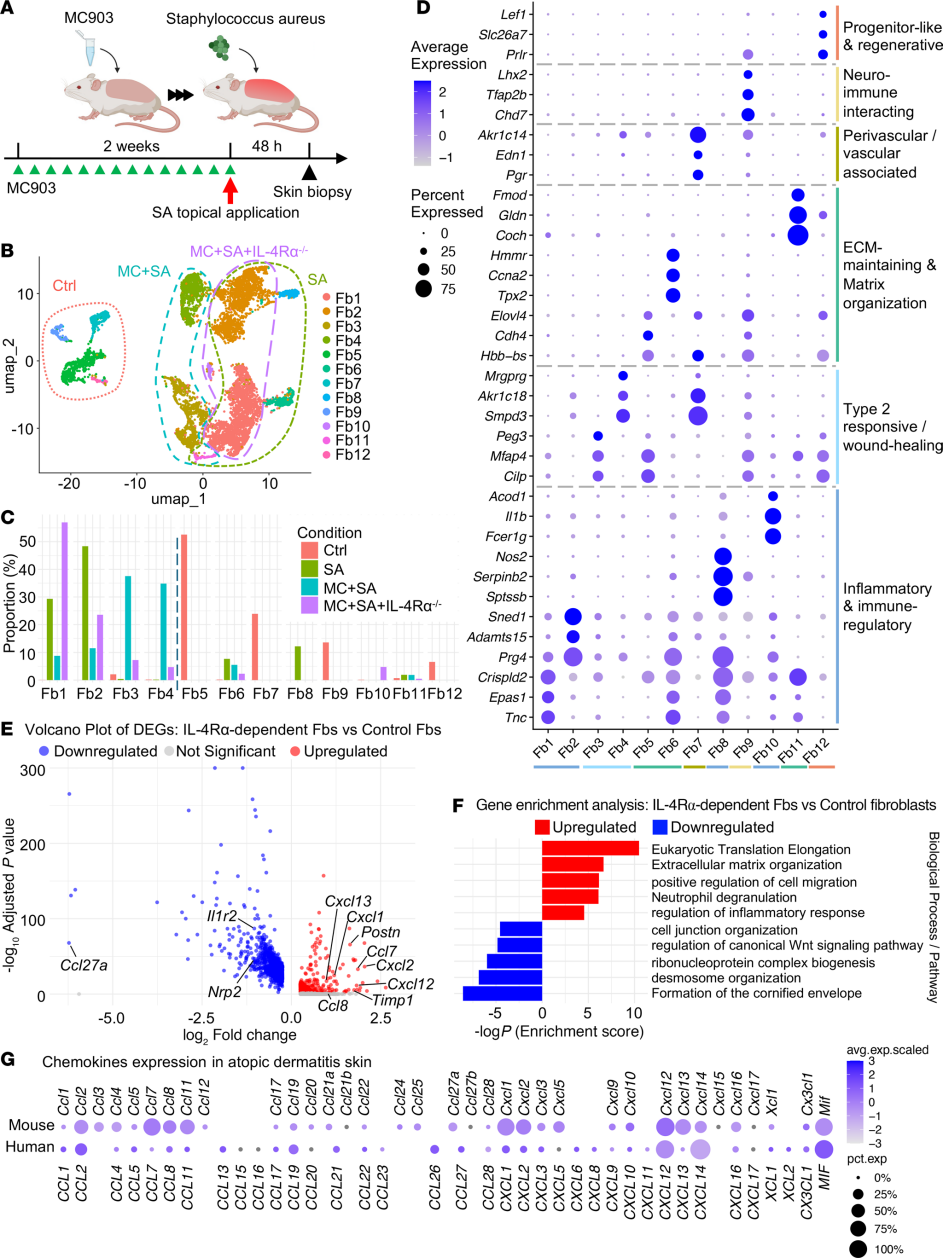
IL-4Rα–dependent dermal fibroblasts express inflammatory chemokines in a mouse model of AD. (**A**) Schematic of murine models of AD following topical application of MC + SA. (**B**) Uniform manifold approximation and projection (UMAP) plot of dermal fibroblast clusters from the back skin of mice in the following groups: control mice (Ctrl), mice treated with SA alone, mice treated with MC + SA, and IL-4Rα^–/–^ mice treated with MC + SA. Dotted circles with each color contain the major cell population in each condition. (**C**) Frequency of each fibroblast cluster in control, SA, MC + SA, and MC + SA + IL-4Rα^–/–^ samples. (**D**) Marker genes associated with specific fibroblast clusters. (**E**) Volcano plot of gene expression comparing IL-4Rα–dependent fibroblast clusters and control fibroblast clusters. (**F**) Gene enrichment analysis for genes upregulated in the IL-4Rα^+^ clusters compared with the control. (**G**) Comparison of chemokine expression profiles in human and mouse dermal fibroblasts in AD skin. avg.exp., average expression; pct.exp, percent expression.

To test for a potential role of dermal fibroblasts in mediating type 2 inflammatory processes such as AD, we evaluated a mouse model of AD. scRNA-seq results from mouse skin following topical application of MC903 (MC) and SA were compared with control mice and mice with systemic loss of the IL-4Rα (Figure 2A) (20). Following cell-type annotation and analysis of chemokine gene expression, we found that several cell types expressed multiple chemokines, but fibroblasts represented the highest proportion of cells expressing multiple chemokines (Supplemental Figure 2, A–D). Intercellular communication analysis by CellChat showed that fibroblast output to T cells is one of the major sources in the T cell input pathways (Supplemental Figure 2, E and F).

Further analysis revealed 12 transcriptionally distinct clusters of dermal fibroblasts in mice (Fb1–Fb12), with distinct patterns of gene expression in several cell types after induction of inflammation by MC, SA, and loss of IL-4 receptor signaling in IL-4Rα^–/–^ mice (Figure 2B and Supplemental Figure 2, G–J). The fibroblast clusters Fb5, Fb7, Fb9, and Fb12 were exclusive to the control skin samples, whereas the remaining clusters appeared in greater frequency in the skin under inflamed conditions (Figure 2C). Fb1 and Fb2 were enriched in control SA mice and in IL-4Rα^–/–^ mice treated with MC and SA (hereafter referred to as MC+SA+IL-4Rα^–/–^ mice), but less prominent in mice treated with MC and SA (hereafter referred to as MC+SA mice). Conversely, Fb3 and Fb4 were most abundant in MC+SA, mice but lost in MC+SA+IL-4Rα^–/–^ mice, suggesting that they represented IL-4– and IL-13–driven fibroblast clusters.

Differentially expressed genes (DEGs) and gene ontology enrichment of 12 mouse dermal fibroblast clusters were analyzed and visualized using dot plots (Figure 2D and Supplemental Figure 2, K–V). Fibroblast clusters were classified into 6 groups on the basis of the major functional and molecular characteristics: inflammatory and immune-regulatory fibroblasts (Fb1, Fb2, Fb8, Fb10), type 2 responsive/wound-healing fibroblasts (Fb3, Fb4), extracellular matrix–maintaining (ECM-maintaining) and matrix-organizing fibroblasts (Fb5, Fb6, Fb11), perivascular- and vascular-associated fibroblasts (Fb7), neuroimmune-interacting fibroblasts (Fb9), and progenitor-like and regenerative fibroblasts (Fb12). The inflammatory and immune-regulatory fibroblast clusters were enriched in SA and MC^+^SA^+^IL-4Rα^–/–^ conditions and expressed genes associated with inflammatory responses and immune cell recruitment, including *Tnc*, *Epas1*, and *Crispld2*. The Fb1 cluster exhibited high expression of *Tnc*, *Epas1*, *Crispld2*, *Cxcl5*, *Mmp3*, and *Timp1*, which are involved in ECM remodeling, inflammatory signaling, and immune cell migration (16, 27–32). The Fb2 cluster exhibited an inflammatory and immune-regulatory profile, with high expression of the genes *Il33*, *Ackr1*, *Ackr3*, *Irak3*, and *Cxcl13*, in addition to ECM-associated genes such as *Prg4*, *Adamts15*, and *Sned1*. This suggests that Fb2 fibroblasts not only share some perivascular features but also act as modulators of immune responses through cytokine and chemokine regulation. Fb8, exclusive to SA-treated skin, had a strong immune-sensing and acute inflammatory signature, characterized by high expression of *Nos2*, *Serpinb2*, and *Sptssb*, together with induction of inflammatory mediators such as *Cxcl2*, *Cxcl3*, *Csf3*, *Lif*, and *Saa1/2*. These genes suggest that Fb8 fibroblasts act as potent amplifiers of neutrophil recruitment and early inflammatory responses upon microbial stimulation. Fb10 displayed a strong proinflammatory and immune-like phenotype, with high expression of *Fcer1g*, *Il1b*, and *Acod1*, together with additional upregulation of inflammatory mediators (*Tnf*, *Ccl3*, *Ccl4*, *Nlrp3*) and myeloid-associated genes (*Cd14*, *Cd68*, *Fcgr3*, *Tyrobp*). These findings suggest that Fb10 fibroblasts adopt an immune-activated program that overlaps with innate immune cell pathways. The type 2 responsive /wound-healing fibroblast group, found predominantly in MC+SA mice, showed IL-4/IL-13–dependent activation. Fb3 exhibited a fibrotic and wound-healing signature, characterized by strong upregulation of ECM and matrix organization genes (*Postn*, *Fbln7*, *Mfap4*, *Col6a1*, *Col15a1*, *Bgn*, *Aspn*) together with fibrotic regulators (*Cilp*, *Peg3*, *Cdh11*). In addition, growth- and migration-related factors (*Mdk*, *Mylk*, *Slit2*, *Pdgfrl*) and Wnt/TGF-β–associated genes (*Sfrp1*, *Dkk3*, *Sox4*) were enriched, supporting its classification as a type 2 responsive/wound-healing fibroblast cluster. Fb4 exhibited a metabolic-inflammatory phenotype, marked by strong expression of metabolic enzymes (*Smpd3*, *Akr1c18*, *Akr1c14*, *Aldh1a3*), together with immune- and stress-responsive genes (*Mrgprg*, *Atf3*, *Socs3*, *Ifi27l2a*). In addition, vascular and ECM remodeling factors (*Tek*, *Sema3c*, *Plxdc2*, *Fn1*) were enriched, suggesting that Fb4 integrates metabolic reprogramming, inflammatory signaling, and vascular remodeling in type 2–driven skin inflammation. The ECM-maintaining and matrix organization fibroblast group, mainly found in control skin, included Fb5 (*Hbb-bs*, *Cdh4*, *Elovl4*), Fb6 (*Tpx2*, *Ccna2*, *Hmmr*), and Fb11 (*Coch*, *Gldn*, *Fmod*), representing fibroblasts specialized in ECM production and structural organization. The perivascular-/vascular-associated fibroblasts in Fb7 expressed *Pgr*, *Edn1*, and *Akr1c14*, together with vascular- and matrix-related genes such as *Itgb7*, *Agtr2*, *Thbd*, and *Plau*, suggesting roles in vascular regulation, barrier support, and local matrix remodeling. The neuroimmune-interacting fibroblasts of Fb9 expressed *Chd7*, *Tfap2b*, and *Lhx2*, along with adhesion and neurodevelopmental genes such as *Itga3*, *Celsr2*, *Casz1*, *Lamb3*, and *Itgb4*, indicating a role in sensory neuron remodeling and immune-neural crosstalk. The progenitor-like and regenerative fibroblasts of Fb12 expressed *Prlr*, *Slc26a7*, and *Lef1*, together with additional stemness and developmental regulator genes such as *Lrrn1*, *Cpne5*, *Tfap2a/c*, *Runx3*, and *Prdm1*, suggesting a regenerative and stem-like fibroblast phenotype. Module score analysis further demonstrated that the vascular/perivascular program was most prominent in Fb7, which was predominantly found in control skin, but appreciable vascular scores were also observed in several other fibroblast clusters (Supplemental Figure 2W). In contrast, the neuroimmune program was largely restricted to Fb9 in the control skin, with only minimal expression detectable in other conditions, suggesting a context-dependent and tightly regulated activity of this cluster. We next examined a follicle/epidermal interface gene set (*Fgf7*, *Rspo3*, *Bmp4*, *Cldn1*, *Corin*, *Alpl*, *Sox18*, *Lef1*, *Coch*) across fibroblast clusters. The *Coch*^+^ signature mapped almost exclusively to Fb11, whereas Fb9 was characterized by *Cldn1*, Fb8 by *Alpl* and *Fgf7*, and Fb12 by *Rspo3*, *Bmp4*, *Corin*, *Sox18*, and *Lef1* (Supplemental Figure 2X). Together with our functional annotations, these data place Fb11 as a Coch-tuned papillary-/appendage-associated fibroblast cluster, Fb9 as an epithelial interface/neuroimmune fibroblast, Fb8 as an appendage-supportive fibroblast cluster, and Fb12 as a progenitor-like follicular program.

The presence of multiple, potentially distinct clusters of dermal fibroblasts is consistent with studies of fibroblasts in other inflammatory conditions and further supports the hypothesis that dermal fibroblasts mediate some cell behaviors during type 2 inflammation (33–35). Notably, although the emergence of certain fibroblast clusters (e.g., Fb3 and Fb4) was dependent on type 2 inflammatory conditions, the expression of *Il4ra* was detected in multiple fibroblast clusters under control conditions and was particularly evident in Fb4 upon stimulation of the skin with MC and SA (Supplemental Figure 2Y).

Next, to better understand the immunological consequences of fibroblast activation during type 2 inflammation, we examined the molecular features of IL-4Rα–dependent fibroblasts (Fb3, Fb4) in inflamed skin. Differential expression analysis comparing IL-4Rα–dependent fibroblasts with homeostatic fibroblasts (Fb5, Fb7, Fb9, and Fb12) revealed upregulation of chemokine genes associated with immune cell migration, including *Ccl7*, *Ccl8*, *Cxcl1*, *Cxcl2*, *Cxcl12*, and *Cxcl13* (Figure 2E). *Ccl7*, *Cxcl1*, *Cxcl2*, *Cxcl12*, and *Cxcl13* were significantly upregulated (adjusted *P* < 0.05, log_2_ fold change [FC] > 0.5), while *Ccl8* showed increased expression but did not reach statistical significance (log_2_ FC = 1.04, adjusted *P* = 1). Positive regulation of cell migration was enriched, aligning with the observed chemokine upregulation. In contrast, pathways associated with cell junction organization, desmosome organization, and canonical Wnt signaling were significantly downregulated (adjusted *P* < 0.05), suggesting alterations in fibroblast adhesion and transcriptional regulation. Additionally, pathways related to ribonucleoprotein complex biogenesis and formation of the cornified envelope were downregulated, indicating a shift away from normal fibroblast differentiation programs (Figure 2F). Compared with the trends of chemokine gene expression in human fibroblasts, although there are species-specific differences, the trends of chemokine expression mouse fibroblast clusters were similar (Figure 2G). These results suggest that fibroblasts are directly responsive to the type 2 immune environment and participate in immune cell recruitment by expressing chemokines.

### IL-4 plus IL-13 directly induce cultured fibroblasts to express chemokines.

**Figure 3:**
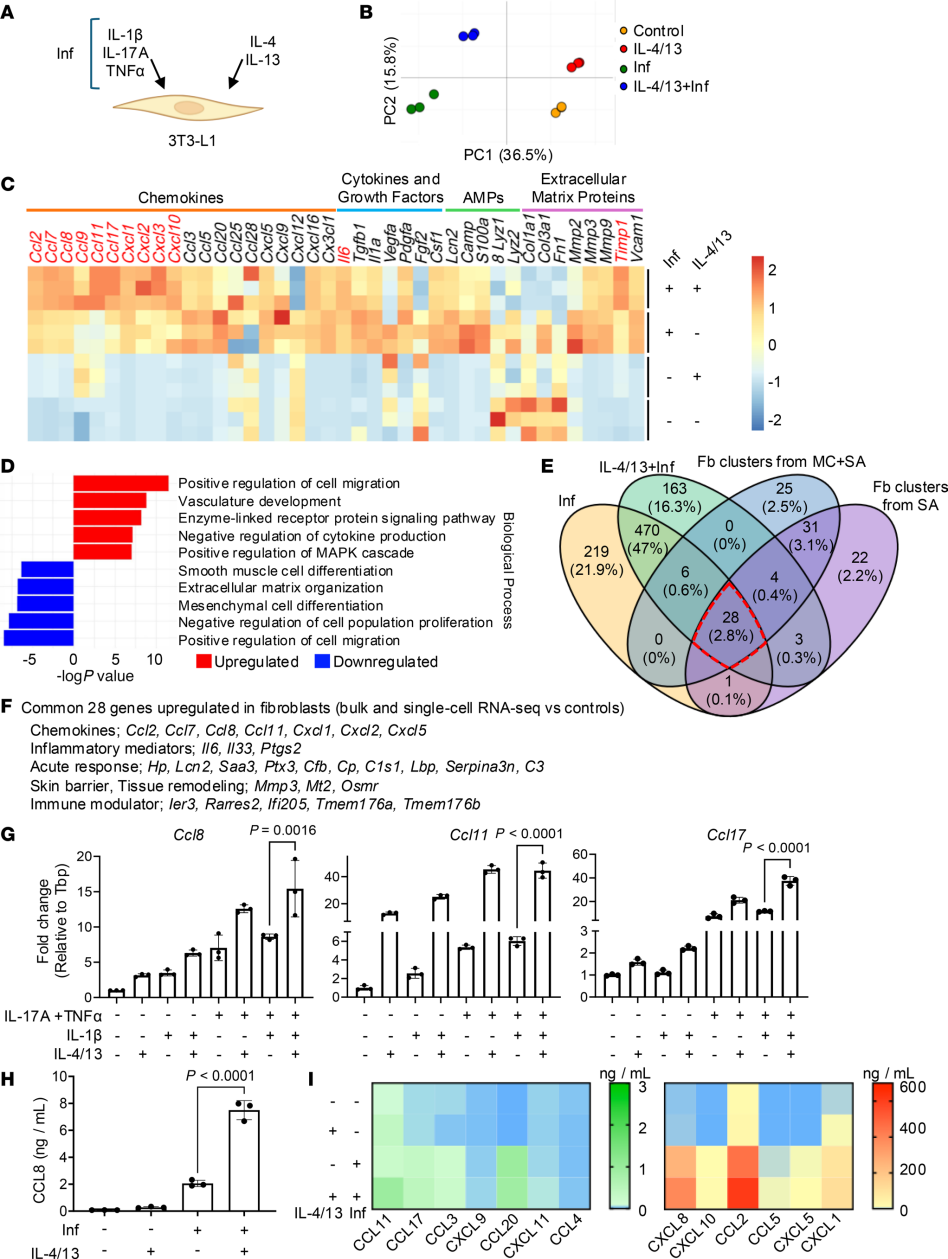
Fibroblasts express multiple chemokines in response to IL-4/13. (**A**) Schematic of the assessment of the response of 3T3-L1 fibroblasts to inflammatory cytokines. (**B**) PCA of the normalized bulk RNA-seq data from fibroblasts 24 hours after culturing under control conditions, with addition of IL-4 (50 ng/mL) and IL-13 (50 ng/mL) (IL-4/13), with the addition of a mixture of inflammatory cytokines TNF-α (20 ng/mL), IL-17A (50 ng/mL), and IL-1β (0.5 ng/mL) (Inf), or with IL-4/13 + Inf. (**C)** Heatmap illustrating changes in selected gene expression, scaled by row. (**D**) Gene enrichment analysis for 3T3-L1 fibroblasts treated with IL-4/13 + Inf and Inf alone. (**E**) Venn diagram showing the overlap of genes induced in cultured fibroblasts and mouse dermal fibroblasts. Shown is the expression of genes induced in cultured fibroblasts following the addition of Inf or IL-4/13 + Inf compared with control culture conditions, and fibroblast gene expression in mice determined by scRNA-seq of mouse skin exposed to SA alone or MC + SA compared with control mouse skin. Numbers indicate the numbers and percentages of upregulated genes with significant differences. The red dashed area highlights the 28 genes commonly upregulated across all 4 conditions. (**F**) List of 28 genes upregulated in fibroblasts in culture or in mice following exposure to type 2 cytokines. (**G**) qRT-PCR analysis of *Ccl8*, *Ccl11*, and *Ccl17* mRNA expression in 3T3-L1 fibroblasts treated with Inf and IL-4/13 as in **B** (*n* = 3). (**H**) Measurement of CCL8 protein concentration in 3T3-L1 fibroblast supernatants by ELISA 72 hours after treatment with Inf or IL-4/13 as in **B** (*n* = 3). (**I**) Heatmap of protein expression measured by LEGENDPLEX in culture supernatants of the human fibroblast cell line HPAd. Statistical significance was determined by 1-way ANOVA followed by Tukey’s multiple-comparison test (**G** and **H**) (*n* = 3). Experiments in **G**–**I** are representative of 2–3 independent experiments. Data are presented as the mean ± SD.

To investigate the direct effect of type 2 cytokines on fibroblasts, we cultured the mouse embryonic fibroblast cell line 3T3-L1 with an inflammatory (Inf) cytokine cocktail of TNF-α, IL-17A, and IL-1β and then evaluated the additional response seen following the addition of IL-4 and IL-13 (IL-4/13) (Figure 3A). This cocktail was chosen because TNF-α, IL-17A, and IL-1β are elevated in human AD (36, 37), and our previous studies demonstrated that TNF-α and IL-17A potently activate dermal fibroblasts to induce chemokines such as CXCL1, CXCL12, and LCN2 (16). The Inf cytokine cocktail was used to replicate the host defense inflammatory skin environment found in AD or induced by injury or SA colonization in mice. To assess the transcriptional responses of fibroblasts under these conditions, we performed bulk RNA-seq analysis under 4 experimental conditions: (a) unstimulated fibroblasts as a control, (b) fibroblasts treated with IL-4/13 alone, (c) fibroblasts stimulated with Inf, and (d) fibroblasts exposed to a combination of IL-4/13 and Inf. Principal component analysis (PCA) of the bulk RNA-seq data revealed distinct transcriptomic profiles for each condition, highlighting the unique transcriptional profile of fibroblasts following the addition of IL-4/13 (Figure 3B). Analysis of DEGs was conducted in each condition relative to the control, and upregulated genes were visualized using a Venn diagram to show that several genes were uniquely expressed by fibroblasts following the addition of IL-4/13 (Supplemental Figure 3A). Fibroblasts cultured in the IL-4/13 + Inf condition showed increased expression of 768 genes compared with control fibroblasts, with 542 genes overlapping with those upregulated by Inf alone. A cluster of 47 genes were commonly upregulated across all 3 cytokine-treated conditions, whereas only 20 genes were shared between IL-4/13 + Inf and IL-4/13, excluding those already induced by Inf. These findings demonstrate that type 2 cytokines were able to directly alter fibroblast gene expression, particularly under inflammatory conditions.

The addition of IL-4/13 to the Inf cytokine environment enhanced the expression of multiple chemokines not induced by IL-4/13 alone and exceeded the expression levels observed under the Inf condition alone (Figure 3C). Notably these included *Ccl2*, *Ccl7*, *Ccl8*, *Ccl11*, *Ccl17*, *Cxcl1*, *Cxcl2*, *Cxcl3*, and *Cxcl10* and the ECM regulator *Timp1* (38). Conversely, and as previously shown in keratinocytes (20), the addition of IL-4/13 decreased the expression of antimicrobial peptide (AMP) genes such as cathelicidin (*Camp*), S100 calcium–binding protein A8 (*S100a8*), and lipocalin 2 (*Lcn2*), compared with Inf alone. Independent DEG analysis found significant changes in the expression of multiple genes by fibroblasts after exposure to IL-4/13, with the top pathway identified as being associated with positive regulation of cell migration (Figure 3D).

To determine whether the expression of genes by fibroblasts in vitro was representative of the response of fibroblasts from the mouse AD model, we compared data from bulk RNA-seq of 3T3-L1 fibroblasts after stimulation with IL-4/13 and Inf with the results of pseudobulk DEG analysis of all dermal fibroblast genes from the in vivo AD mouse. We found that 28 genes were upregulated in both fibroblasts from mice after a type 2 inflammatory response and in cultured fibroblasts exposed to IL-4/13 + Inf (Figure 3, E and F). Several of these genes (*Ccl2*, *Ccl7*, *Ccl8*, *Ccl11*, *Cxcl1*, *Cxcl2*, and *Cxcl5*) were also expressed in dermal fibroblasts from individuals with AD (Figure 2G). These results suggest a conserved fibroblast transcriptional program across in vivo and in vitro type 2 inflammatory settings and support the relevance of the mouse as a model for studying the role of fibroblasts in human AD.

Next, we performed quantitative reverse transcription PCR (qRT-PCR) to quantitatively validate the results obtained by RNA-seq. These results confirmed that the addition of IL-4/13 to IL-17A + TNF-α, or to the Inf cytokine cocktail of IL-17A + TNF-α + IL-1β, significantly upregulated the expression of mRNA for C-C motif chemokines (CC chemokines) in 3T3-L1 fibroblasts (Figure 3G and Supplemental Figure 3B). We observed a similar pattern of induction for *Ccl2* and *Ccl7* in mouse primary dermal fibroblasts, although *Ccl8* decreased upon the addition of IL-4/13 to Inf, potentially reflecting the heterogeneous nature of primary cell culture containing multiple transcriptionally distinct fibroblasts (Supplemental Figure 3C). CCL8 protein increased in 3T3-L1 fibroblast culture supernatant after activation by IL-4/13 + Inf compared with Inf alone (Figure 3H). The human-derived fibroblast cell line HPAds showed increased expression of multiple chemokine proteins (Figure 3I) and induction of mRNA (Supplemental Figure 3D) after activation by IL-4/13 + Inf, thus demonstrating that the direct response to IL-4/13 also occurs in the human system.

On the basis of the gene sets induced in 3T3-L1 cells by Inf, IL-4/13, and IL-4/13 + Inf, we generated module scores for each fibroblast cluster identified from mouse skin by scRNA-Seq (Supplemental Figure 3E). Violin plot analysis demonstrated that the module score was highest across multiple fibroblast subclusters when generated from 3T3-L1 cells activated by Inf. Notably, the module score of Fb4 was highest when determined by comparison with 3T3-L1 cells activated by the addition of IL-4/13 + Inf. This aligns with the cluster enriched in the mouse AD model when comparing WT mice with IL-4Rα^–/–^ mice. This alignment further indicates that the 3T3-L1 cell line can model some fibroblast clusters in vivo that are responsive to IL-4/13.

### IL-4/13 enhance the capacity of fibroblasts to promote T cell migration by chemokines recognized by CCR3.

**Figure 4:**
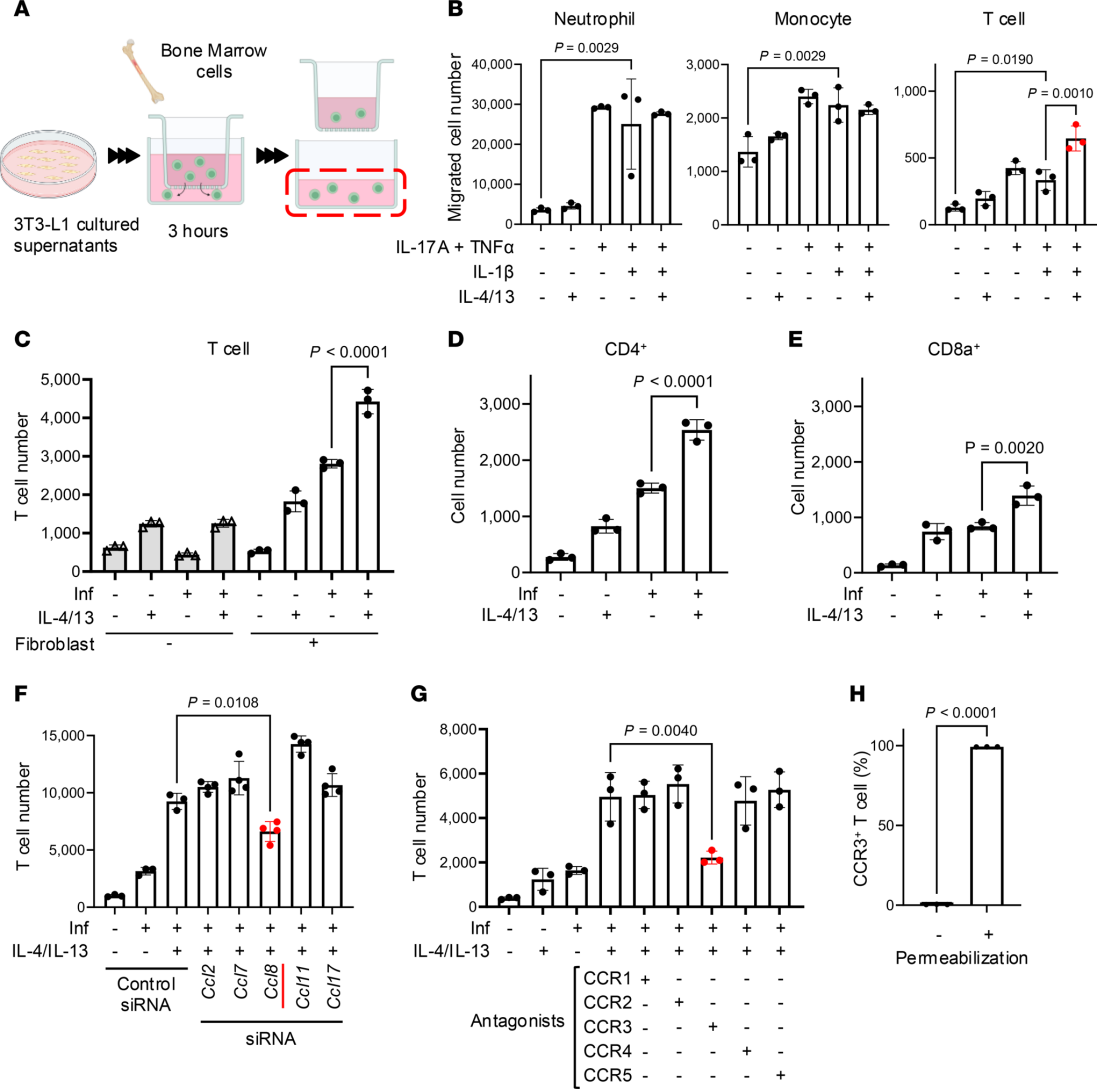
Fibroblasts recruit T cells through the production of multiple chemokines. (**A**) Schematic of the migration assay assessing the chemotactic activity of culture supernatant from fibroblasts. (**B**) Number of mouse bone marrow cells migrated toward the lower chamber containing fibroblast cultured supernatant. (**C**) Number of CD3e^+^ T cells from mouse splenocytes that migrated toward the lower chamber containing fibroblast cultured supernatant. (**D**) Comparison of the number of CD4^+^ T cells from the splenocyte migration assay. (**E**) Comparison of CD8a^+^ T cell numbers from the migration assay. (**F**) Comparison of CD3e^+^ T cell numbers in culture supernatants of fibroblasts after the indicated chemokine siRNA treatments. (**G**) Comparison of CD3e^+T^ cell numbers after pretreatment with the indicated CCR antagonists in migration assays toward fibroblast culture supernatants. (**H**) Percentage of CD3e^+^ T cell staining for CCR3 before and after cell permeabilization. Statistical significance was determined by 1-way ANOVA followed by Tukey’s multiple-comparison test (**B**–**G**) or unpaired, 2-tailed Student’s *t* test (**H**) (*n* = 3). Experiments in **B**–**H** are representative of 2–3 independent experiments. Data are presented as the mean ± SD.

To determine whether the secretome of fibroblasts is sufficient to induce cell migration, we next performed a cell migration assay using the culture supernatant of fibroblasts activated by IL-4/13 and inflammatory cytokines (Figure 4A). The culture supernatant of fibroblasts stimulated with TNF-α and IL-17A greatly increased the migration of neutrophils, monocytes, and T cells compared with the culture supernatant of unstimulated fibroblasts, but the addition of IL-4/13 alone did not (Figure 4B). IL-1β did not further enhance the activity of TNF-α and IL-17A to induce fibroblasts to release chemotactic activity under these conditions. However, the addition of IL-4/13 + Inf to fibroblasts selectively promoted T cell migration from the mixed immature cell population prepared from mouse bone marrow. Cell migration assays performed with mouse splenocytes showed a similar response, demonstrating that fibroblasts stimulated by IL-4/13 significantly promoted T cell migration (Figure 4C and Supplemental Figure 4A). Both CD4^+^ and CD8a^+^ T cell populations were recruited by the supernatant of fibroblasts activated by IL-4/13 + Inf (Figure 4, D and E, and Supplemental Figure 4B), but control experiments done with media containing IL-4/13 + Inf without fibroblasts did not promote significant cell migration.

Next, to determine which chemokines induced by IL-4/13 in fibroblasts were responsible for the capacity of fibroblasts to promote T cell migration, we performed siRNA knockdown of *Ccl2*, *Ccl7*, *Ccl8*, *Ccl11*, and *Ccl17* in 3T3-L1 fibroblasts and tested the cultured supernatants individually in the migration assay. siRNA knockdown of *Ccl8* was most effective in inhibiting T cell migration (Figure 4F and Supplemental Figure 4C). Furthermore, analysis of the capacity of a panel of chemokine receptor inhibitors showed that the CCR3 antagonist SB-328437 was most effective in inhibiting T cell migration driven by fibroblasts (Figure 4G and Supplemental Figure 4D). Permeabilization confirmed that CCR3 was present in the splenocyte population (Figure 4H and Supplemental Figure 4E).

### CCR3 is critical for T cell recruitment and inflammation in a mouse model of AD.

**Figure 5:**
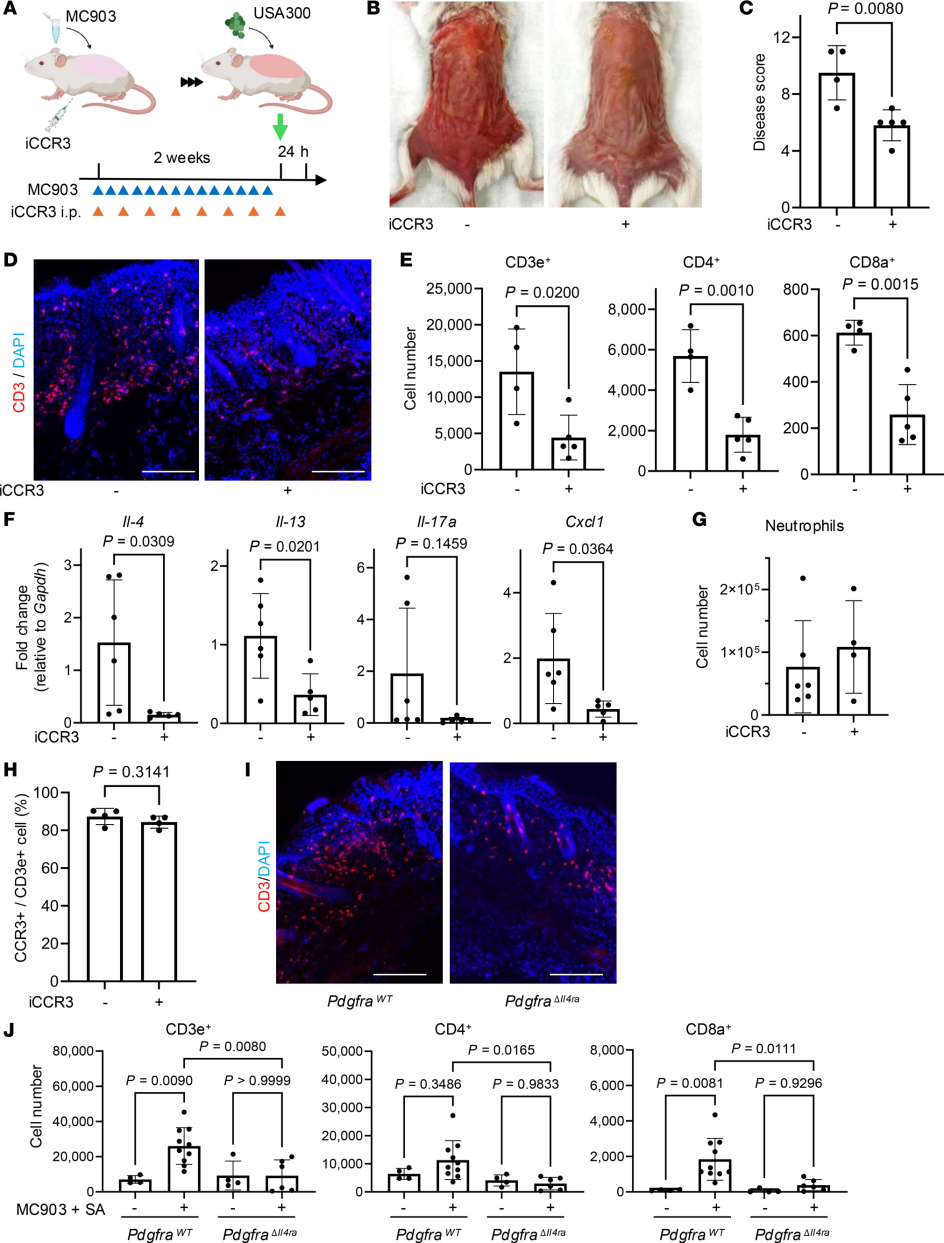
CCR3 antagonist reduces T cell numbers in skin and AD-related cytokine expression. (**A**) Schematic of mouse model of AD treated with CCR3 antagonist (iCCR3, SB-328437, 20 μg/g body weight, administered i.p. every other day). (**B**) Representative images of dorsal skin from mice treated as in **A**, with and without iCCR3. (**C**) Disease score for mice treated as in **A**, with and without iCCR3. (**D**) Immunofluorescence images of CD3^+^ T cells (red) and nuclei (DAPI, blue) in dorsal skin from mice treated as in **A**, with and without iCCR3. Scale bars: 300 μm. (**E**) Flow cytometric quantification of CD3e^+^, CD4^+^, and CD8a^+^ T cells in dorsal skin from mice treated as in **A**, with and without iCCR3. (**F**) qRT-PCR analysis of *Il4*, *Il13*, *Il17a*, and *Cxcl1* mRNA expression in dorsal skin from mice treated as in **A**, with and without iCCR3. (**G**) Flow cytometric quantification of neutrophils in dorsal skin from mice treated as in **A**, with and without iCCR3. (**H**) Ratio of CCR3^+^ cells of CD45^+^CD3e^+^ cells in dorsal skin from mice treated as in **A**, with and without iCCR3. (**I**) Immunofluorescence images of CD3^+^ T cells (red) and nuclei (DAPI, blue) in dorsal skin from *Pdgfra*^ΔIl4ra^ mice treated as in **A**. Scale bars: 300 μm. (**J**) Flow cytometric quantification of CD3e^+^, CD4^+^, and CD8a^+^ T cells in dorsal skin from *Pdgfra*^ΔIl4ra^ and control mice treated in the MC + SA model. Statistical significance was determined by unpaired, 2-tailed Student’s *t* test (**C** and **E**–**H**) or 1-way ANOVA followed by Tukey’s multiple-comparison test (**J**) (*n* = 3–10). Results are representative of 2–3 independent experiments. Data are presented as the mean ± SD.

Having demonstrated that dermal fibroblasts are a major source of chemokines that recruit T cells and that CCR3 is important to this process, we next measured the response to inhibition of CCR3 in mice by administration of a CCR3 antagonist (iCCR3, SB-328437, MedChemExpress) via i.p. injection (Figure 5A). Administration of iCCR3 to mice after MC + SA decreased redness and reduced disease scores (Figure 5, B and C). Histological analysis showed decreased CD3^+^ cells in the dermis and reduced skin thickness after iCCR3 treatment (Figure 5D and Supplemental Figure 5A). Flow cytometric analysis confirmed a significant reduction in total T cell numbers, including both CD4^+^ and CD8^+^ T cells after iCCR3 treatment in the AD model (Figure 5E). To determine whether iCCR3 similarly inhibits T cell migration in an alternative, allergic model of AD in mice, we also assessed mice after sensitization with OVA and topical SA (Supplemental Figure 5B) (39). Consistent with the results observed in the MC + SA model, we found that iCCR3 treatment reduced CD3^+^ T cell infiltration in the dermis (Supplemental Figure 5C), and flow cytometric analysis revealed a significant decrease in T cells (Supplemental Figure 5D). Across both AD models, iCCR3 treatment was associated with a trend toward decreased expression of inflammatory cytokine gene expression, including *Il4*, *Il13*, *Il17a*, and *Cxcl1* (Figure 5F and Supplemental Figure 5E). No significant decrease in neutrophils present in the skin was seen under either condition after iCCR3 treatment (Figure 5G and Supplemental Figure 5F). Furthermore, costaining for CCL8 and PDGFRα in mouse AD skin showed that CCL8 protein was expressed in the epidermis as well as in Pdgfra^+^ fibroblasts in the deep dermis (Supplemental Figure 5G). Flow cytometric analysis of skin-infiltrating CD45^+^CD3^+^ cells demonstrated that 80% of the CD3^+^ T cells expressed CCR3 (Figure 5H). Since these CD3^+^ T cells were primarily localized in the dermal compartment adjacent to the CCL8 protein expressed by dermal fibroblasts and the numbers of these CD3^+^ T cells significantly decreased in skin after iCCR3 treatment (Figure 5, D and E), these findings suggest that a spatial association between fibroblast CCL8 and CCR3^+^ T cells is functionally relevant.

### Dermal fibroblasts are important for the recruitment of T cells in mice during type 2 inflammation.

To specifically investigate the role of fibroblast IL-4Rα in T cell recruitment during type 2 inflammation, we next tested mice with a deletion of IL-4Rα targeted to fibroblasts by crossing *Pdgfra*-Cre mice with *Il4ra^fl/fl^* mice (*Pdgfra^ΔIl4ra^*). Histological analysis revealed that *Pdgfra^ΔIl4ra^* mice had decreased CD3^+^ cell frequencies in the deeper dermis (Figure 5I), a pattern consistent with the predominant expression of CCL8 seen on deep reticular fibroblasts, as shown in Supplemental Figure 5G. Flow cytometric analysis also showed a significant reduction of total T cell numbers, including both CD4^+^ and CD8^+^ T cells, in the skin of *Pdgfra^ΔIl4ra^* mice following application of MC + SA (Figure 5J).

## Discussion

In this study, we demonstrate that dermal fibroblasts have distinct immunological functions in the context of AD and play an important role in driving T cell recruitment. An integrative analysis of scRNA-seq data from both human and murine skin revealed the induction of chemokines by distinct fibroblast clusters in a type 2 inflammatory environment. Further testing of cultured fibroblasts from mice and humans confirmed the induction of chemokines observed in the scRNA-seq data from AD skin and showed that the capacity of IL-4 and IL-13 to directly promote T cell recruitment was dependent on their action in a background inflammatory environment modeled in vitro by additional activation of fibroblasts by TNF-α, IL-17A, and IL-1β. In mice, the specific role of fibroblasts in promoting T cell recruitment in a model of AD was demonstrated by targeted deletion of IL-4Rα in fibroblasts. Additionally, since the major chemokines induced by dermal fibroblasts are ligands of CCR3 (40, 41), we demonstrate that use of a selective CCR3 inhibitor effectively inhibited T cell recruitment both in vitro and in mice. These findings reveal a previously underappreciated role for fibroblasts as important mediators of the inflammatory response seen in AD.

In this study, a role for fibroblasts in AD was enabled by unbiased scRNA-seq analyses, which identified several transcriptionally distinct fibroblast clusters that showed increased chemokine expression under type 2 inflammatory conditions in both human and mouse AD skin. Network analysis further revealed that fibroblasts likely participate as a hub for cell communication with inflammatory cells to mediate cell recruitment during AD. To functionally assess this, we tested how cultured mouse or human fibroblasts respond to IL-4/13 alone, or in the presence of an Inf cytokine cocktail of TNF-α, IL-17A, and IL-1β. This Inf cocktail was included in our experiments, since these factors are also elevated in AD and have been independently observed to activate fibroblast chemokine release and neutrophil recruitment (16, 17). Thus, the addition of the cytokine cocktail to IL-4/13 more closely modeled the type 2 inflammatory environment than addition of IL-4/13 alone. Under this experimental design, we observed that IL-4 and IL-13 had minimal activity alone, but markedly increased the gene expression of chemokines such as *Ccl2*, *Ccl7*, *Ccl8*, and *Ccl17* when added to the Inf cytokine cocktail. These results suggest that type 2 cytokines alter and amplify fibroblast chemokine expression, thus revealing a type 2 transcriptional shift by fibroblasts activated to participate in inflammation, a transcriptionally distinct state we have previously described as immune-acting fibroblasts (IAFs) (16, 17). The altered expression of chemokines following the addition of IL-4 and IL-13 to IAFs correlated with an increase in recruitment of both CD4^+^ and CD8^+^ T cells in vitro, while no further recruitment of neutrophils or monocytes was observed. Thus, IAFs appear to need to achieve a state of inflammatory activation for optimal T cell chemotactic activity. While the results from in vitro cell migration do not necessarily translate to cell recruitment in AD, they do suggest that further analysis of the role of dermal fibroblasts and other potential activating events is needed. Furthermore, it highlights the potential benefit of combination therapy for some patients with AD who experience resistance to monotherapy directed only against type 2 cytokines.

Observations of T cell migration with fibroblast-conditioned media suggest that no single factor is uniquely responsible for this function, although CCL8 had the most pronounced effect. Pharmacologic inhibition and siRNA-mediated knockdown of chemokine receptors identified CCR3 as likely to mediate this response. Notably, as previously reported (40–43), CCR3 surface expression on T cells was low and not detectable by scRNA-seq, but was abundantly detectable following permeabilization, suggesting that this receptor cycled rapidly from the surface to an intracellular localization, where it accumulated at the highest concentration, thus limiting the use of scRNA-seq to detect this communication pathway. Furthermore, another limitation in defining the significance of the CCR3 axis in AD was that, while our migration assays used mouse splenic and bone marrow–derived T cells, the migratory behavior of T cells in human skin may differ (44). This underscores the need for further in situ studies to evaluate T cell–fibroblast interactions directly within the human AD skin microenvironment.

To test the physiological relevance of fibroblasts during type 2 inflammation in vivo, we used 2 AD mouse models — MC + SA or OVA + SA (20, 39). We found that pharmacologic inhibition of CCR3 markedly reduced skin inflammation and T cell infiltration in both settings. *Il4*, *Il13*, and *Cxcl1* gene expression was also reduced, whereas neutrophil infiltration remained unchanged. These findings were consistent with our observations of the difference in gene expression between fibroblasts exposed to TNF-α and IL-17A alone or with the addition of IL-4/13 and support a role for CCR3 in T cell–mediated skin inflammation, as well as its well-known activity in eosinophil recruitment (45). However, CCR3 is also expressed in many cell types, including other fibroblast clusters, mast cells, basophils, and DCs (46–54). The systemic administration of the CCR3 antagonist will likely influence these other cell populations. Similarly, ligands for CCR3 are produced by other cell types, and our findings do not conclusively demonstrate that chemokines derived from dermal fibroblasts are the source targeted by the antagonist. However, we show in Figure 1A that dermal fibroblasts were the most abundant cell type expressing chemokines that bind CCR3, such as *CCL5*, *CCL7*, *CCL8*, *CCL11*, *CCL13*, *CCL14*, *CCL24*, and *CCL26* (55–62). Therefore, the improved phenotype after inhibition of CCR3 was likely due in large part to fibroblast-derived C-C motif chemokine ligands (CCLs).

To specifically investigate the relative contribution of fibroblasts to T cell recruitment during type 2 inflammation, we tested the response of mice with targeted deletion of IL-4Rα in fibroblasts by crossing *Pdgfra*-Cre mice with *Il4ra^fl/fl^* mice (63). *Pdgfra*-Cre has previously been used by us to selectively and effectively delete an IL-17 receptor in fibroblasts (16, 17). We found that deletion of IL-4Rα on dermal fibroblasts markedly reduced T cell infiltration in the MC mouse model of AD, with major loss of T cells observed in the deep reticular dermis, the predominant location of IAFs identified in other inflammatory skin models (16, 17). These findings support the conclusion that IL-4Rα signaling in dermal fibroblasts is an important factor for T cell recruitment during type 2 inflammation and that the reduction of chemokine expression in fibroblasts may influence only the local microenvironment, rather than the entire skin, as it could be offset by a compensatory increase in other cell lineages.

While these observations provide important and previously unappreciated insight into the role of fibroblasts in AD, some limitations must be acknowledged. Transcriptomics analysis by scRNA-seq will underrepresent rare cells, low-abundance transcripts including *Ccr3*, and fragile cell types such as granulocytes and adipocytes (40, 64, 65). To adjust for this, we complemented these findings with studies using cultured fibroblasts to validate some observations. We recognize that fibroblast-like cell lines, as well as cultures of primary dermal fibroblasts, do not fully replicate the behavior of fibroblasts in vivo and that these likely reflect only some of the IAF clusters observed by scRNA-seq. Additional work with isolated dermal fibroblasts reflective of the different clusters is warranted. This, however, is difficult, as prior studies of fibroblast isolates show these cells to be highly plastic (66), suggesting that the transcriptionally distinct fibroblast populations are cells captured at one activation state in time.

In conclusion, this study reveals that dermal fibroblasts are likely to actively contribute to the pathogenesis of AD by responding to IL-4/13 and producing chemokines that engage CCR3 on T cells, thereby promoting their recruitment into inflamed skin. This finding challenges the long-standing notion of fibroblasts as passive bystanders and suggests that fibroblast-targeted modulation of immune pathways, including chemokine signaling, may represent a promising therapeutic strategy for AD. By illuminating the immunological functions of fibroblasts, our findings expand the current understanding of stromal-immune cell interactions in chronic skin inflammation. This work can guide future efforts in developing localized, cell-specific interventions for allergic skin disease.

## Methods

### Sex as a biological variable.

Both male and female mice were used for all in vivo experiments. To our knowledge, no sex-specific differences in the responses to MC, SA, or OVA-induced inflammation have been reported in these models, and no sex-based differences were observed in our preliminary analyses. Therefore, data for both sexes were pooled for all experiments. The findings are expected to be applicable to both sexes.

### Study design.

This study was designed to test the hypothesis that dermal fibroblasts contribute to the skin inflammatory response in AD. We reanalyzed scRNA-seq data from human (25) and mouse (20) skin to focus on fibroblast immune responses. Functional validation was conducted through in vitro cytokine stimulation, cell migration assays, and in vivo experiments using murine AD models.

### Animal experiments.

BALB/c and C57BL/6 mice (6–8 weeks old, both sexes; The Jackson Laboratory) were maintained under specific pathogen–free conditions with a 12-hour light/12-hour dark cycle at 20°C–22°C and 30%–70% humidity.

*Flg^fl/fl^* BALB/c mice were provided by Raif S. Geha (Boston Children’s Hospital), as previously described (39). *Pdgfra*^ΔIl4ra^ mice (C57BL/6J background) were provided by Michael D. Rosenblum (UCSF).

A mouse model of type 2 inflammation with topical application of the vitamin D analog MC followed by topical application of SA was used with some modifications of the previously described procedure (20). Briefly, the dorsal skin of BALB/c mice was shaved, depilated, and topically treated with 45 μM MC (50 μL in ethanol) daily for 14 days. On the final day, if indicated, a TSB agar disc (3 × 2 cm) containing 3.0 × 10^6^ CFU of SA USA300 (ATCC BAA-1717) was applied to the skin and covered with Tegaderm (3M Health Care [Solventum]) for 24 hours. A second model of type 2 inflammation in mice made use of *Flg^fl/fl^* BALB/c mice that were sensitized i.p. with OVA (100 μg/100 μL in 50% alhydrogel) once a week for 3 weeks (67). After shaving, the dorsal skin was challenged with a sterile patch (3 × 2 cm) soaked with OVA (600 μg/600 μL in PBS) and covered with Tegaderm. Patches were replaced daily for 6 days. After the challenge period, SA agar discs were applied as described above.

To investigate the effect of CCR3 in these models, the CCR3 antagonist SB-328437 (MedChemExpress, HY-103363) was dissolved at 200 mg/mL in DMSO (100 mg in 500 μL) (68, 69). The solution was diluted with PEG300 and PBS to achieve 2% DMSO, and i.p. injections of 400 μg per mouse (equivalent to 20 μg/g body weight for a 20 g mouse) were administered every other day during the AD induction period in both models.

To reveal the specific contribution of dermal fibroblasts to T cell infiltration during type 2 inflammation, experiments were also performed using *Pdgfra^ΔIl4ra^* mice on a C57Bl6J background.

### scRNA-seq and analysis.

Human scRNA-seq data were obtained from the GEO database (GEO GSE147424), and mouse scRNA-seq data were reanalyzed from a previously published study (20). All datasets were processed using Seurat (version 5.3.0) for normalization, dimensionality reduction, clustering, and cell-type annotation based on canonical marker genes. To correct for sample-level batch effects within each species, the Harmony algorithm (version 1.2.3) was applied following PCA (70). For human datasets, default parameters were used with 10 principal components. For mouse datasets, Harmony was run using 15 principal components with adjusted parameters (theta = 3, lambda = 0.5, max.iter = 100) to account for increased biological variability across multiple treatment conditions. Harmony embeddings were used for downstream clustering and visualization. Chemokine gene expression and intercellular communication were analyzed using the CellChat package (version 1.6.1) (71).

### Human skin scRNA-seq data preprocessing.

Raw gene expression matrices (.txt.gz) were downloaded from the GEO database (GEO GSE147424). Files were parsed and cleaned to remove extraneous characters, and sample identities were assigned to 1 of 3 conditions: healthy control (HC), nonlesional atopic dermatitis (NL), or lesional atopic dermatitis (LS). Gene-by-cell matrices were aligned across samples by appending zero-filled rows for missing genes and then merged into a single expression matrix.

Cells were retained for downstream analysis if they met all of the following quality control (QC) criteria: (a) number of detected genes >200 and <5,000; (b) mitochondrial gene content <20%; (c) erythrocyte-associated gene content <10%; and (d) ribosomal gene content <40%. Cells failing these thresholds were excluded. The RNA assay was used directly without global normalization to preserve the relative transcript abundance for integration and batch correction.

### Mouse skin scRNA-seq data preprocessing.

Dorsal skin samples were collected from 4 experimental conditions: (a) WT_control: vehicle-treated WT mice; (b) WT_SA: WT mice topically treated with SA; (c) WT_MC_SA: WT mice topically treated with MC + SA; and (d) IL4KO_MC_SA: IL-4 receptor–deficient mice topically treated with MC + SA.

Single-cell suspensions were generated and processed using Read10X() in Seurat. To remove ambient RNA contamination, we applied the decontX() function from the celda package (version 1.14.0) to each sample after conversion to SingleCellExperiment objects. Decontaminated count matrices (decontXcounts) were subsequently used to create Seurat objects.

Ribosomal genes (defined as gene names beginning with “^Rps” or “^Rpl”) were removed from each dataset. Mitochondrial gene content was calculated using PercentageFeatureSet() with the pattern “^mt-”. Cells were retained if they satisfied the following QC criteria: (a) number of detected genes >200 and <6,000; (b) total UMI counts >1,000 and <40,000; and (c) mitochondrial gene content <20%.

Filtered Seurat objects were saved and used for downstream integration, clustering, and analysis.

### Cell-type identification.

For both human and mouse datasets, dimensionality reduction and clustering were performed using Seurat (version 5.3.0). After QC filtering and normalization (as described above), highly variable features were identified, and PCA was applied. Batch correction using Harmony (version 1.2.3) was performed with the sample metadata (group.by.vars = “sample” for human, “orig.ident” for mouse). Clustering was conducted using the “FindNeighbors()” and “FindClusters() functions,” and UMAP was used for 2D visualization. Cell types, including fibroblasts, keratinocytes, myeloid cells, T cells, endothelial cells, pericytes, sweat gland cells, mast cells, melanocytes, and sebaceous gland cells, were annotated according to canonical marker gene expression. Annotation accuracy was manually confirmed using DotPlot and FeaturePlot visualizations.

The full lists of DEGs across cell-type clusters for both human and mouse datasets are shown in the Supporting Data Values file.

### Pseudobulk differential expression analysis.

To complement single-cell-level comparisons, fibroblast pseudobulk profiles were generated by summing raw counts across all fibroblasts within each sample. This resulted in a gene × sample count matrix that preserved sample-level biological replication. Differential expression was performed using edgeR with trimmed mean of M-values (TMM) normalization. Pairwise exact tests were conducted between the control group and each treatment group (SA; MC + SA; MC + SA + IL-4Rα^–/–^), and genes with a FDR of less than 0.05 and a log_2_ FC of greater than 0.25 were considered significantly upregulated. For visualization, pseudobulk counts were transformed to counts per million (CPM), log_2_-transformed, and *z* score–normalized per gene. For integration with bulk RNA-seq data from in vitro–stimulated 3T3-L1 fibroblasts, differential expression was analyzed using DESeq2 between the untreated and each condition. Ensembl IDs were converted to gene symbols via biomaRt, and overlaps between pseudobulk fibroblast DEGs and bulk RNA-seq DEGs were visualized with Venn diagrams (ggvenn, VennDiagram). This approach allowed for direct comparison of cytokine-induced transcriptional programs in vitro with fibroblast responses observed in vivo.

### Differential expression and intercellular communication in all cell types.

DEG analysis was performed using Seurat’s FindMarkers() function (Wilcoxon rank-sum test, Bonferroni-adjusted *P* < 0.05, log_2_ FC >0.25). Visualization was done using ggplot2-based dot plots merged with bar plots for each cell type proportion. Intercellular communication networks were inferred using CellChat (version1.6.1). The “identifyOverExpressedGenes(),” “computeCommunProb(),” and “netAnalysis_signalingRole()” functions were used to calculate ligand-receptor interactions and their biological roles. Communication weights were defined as the communication probability scores calculated by CellChat, which integrate the average expression levels of ligands and receptors with the proportion of cells expressing these genes in each cluster, followed by permutation testing to control for background expression. All analyses were performed separately for each species and condition.

### Fibroblast stimulation experiments.

Mouse 3T3-L1 and human HPAd fibroblasts were stimulated with inflammatory cytokines (TNF-α, IL-1β, IL-17A) with or without IL-4/13. Gene expression was analyzed by bulk RNA-seq and qRT-PCR, and protein expression in fibroblasts cultured supernatants was assessed using ELISA and the LEGENDplex Human Proinflammatory Chemokine Panel 1 (13-plex).

### Transwell migration assays.

Transwell migration assays were performed using culture supernatants from cytokine-stimulated fibroblasts after 0.22 μm filtering (SLGVR33RS, Millex-GV, PVDF membrane, Merck). Immature bone marrow–derived cells or splenocyte-derived T cells were applied to assess chemotactic activity in cell migration chambers (5 μm 24-well Transwell plate; 3421, Coster). siRNA knockdown was performed on 80%–90% confluent 3T3-L1 fibroblasts. Transfection was performed using RNAi Max Lipofectamine (13778075, Thermo Fisher Scientific) and OptiMEM (31985-062, Gibco, Thermo Fisher Scientific) according to the manufacturer’s protocol. siRNAs were used at 15 or 30 nM and included Silencer Negative Control no. 1 siRNA (AM4611, Invitrogen, Thermo Fisher Scientific) as a nontargeting control and siRNAs targeting *Ccl2* (L-042243-00-0005, Dharmacon), *Ccl7* (s73448, Thermo Fisher Scientific), *Ccl8* (AM16708, Thermo Fisher Scientific), *Ccl11* (s73413, Thermo Fisher Scientific), and *Ccl17* (s73420, Thermo Fisher Scientific). Chemokine receptor antagonists targeting the CC receptors mentioned below were preincubated with mouse splenocytes on ice for 30 minutes prior to migration assay for receptor blockade: CCR1 inhibitor (BX471, 0.1 μg/mL; HY-12080, MedChemExpress); CCR2 inhibitor (CAS 445479-97-0, 10 μg/mL; sc-202525, Santa Cruz Biotechnology); CCR3 inhibitor (SB-328437, 10 μg/mL; HY-103363, MedChemExpress); CCR4 inhibitor (C-021, 0.1 μg/mL; HY-103364, MedChemExpress); and CCR5 inhibitor (Maraviroc, 10 μg/mL; HY-13004, MedChemExpress).

### Histological and flow cytometric analysis.

Skin tissues were embedded, unfixed, in O.C.T. compound (4585, Thermo Fisher Scientific) and stored at –80°C. Cryosections were prepared and stained with H&E or subjected to immunofluorescence staining using antibodies against CD3e (14-0032-82, Thermo Fisher Scientific), CCL8 (bs-1985R, Bioss Antibodies), and Gr-1 (108435, BioLegend). Single-cell suspensions from skin were analyzed by flow cytometry to quantify T cell and neutrophil infiltration. Single-cell suspensions were first stained with a viability dye (Aqua Live/Dead Stain Kit, L34957, Thermo Fisher Scientific) and incubated with anti–mouse CD16/CD32 (14-0161-85, eBioscience) to block Fc receptors. Surface staining was subsequently performed using a fluorochrome-conjugated antibody cocktail against CD45 (PE-Texas Red, MCD4517, Invitrogen, Thermo Fisher Scientific), Ly6G (Alexa Fluor 700, 56-5931-82, Invitrogen, Thermo Fisher Scientific), CD11b (APC/Cyanin7, 101226, BioLegend), CD3e (PE, 12-0031-82, Invitrogen, Thermo Fisher Scientific), CD4 (FITC, 11-0042-82, Invitrogen, Thermo Fisher Scientific), CD8a (Brilliant Violet 605, 100743, BioLegend), CD25 (Pacific Blue, 102021, BioLegend), and CCR3 (PercP/Cyanin5.5, 144515, BioLegend). After surface staining, cells were fixed using Stabilizing Fixative Buffer (664907, BD Biosciences). Intracellular staining for CCR3 was performed after permeabilization with permeabilization buffer (00-8333-56, Invitrogen, Thermo Fisher Scientific). Flow cytometric acquisition was performed on a BD FACSCanto RUO flow cytometer, and data were analyzed using FlowJo software (version 10).

### Disease symptom scoring.

Clinical skin disease scores were assessed 24 hours after the topical application of SA to the dorsal skin of mice. The following 4 clinical parameters were evaluated separately using a semiquantitative scale: erythema, edema, erosion or skin disruption, and lichenification. The severity of each parameter was scored as follows: 0 for no symptoms, 1 for mild symptoms, 2 for moderate symptoms, and 3 for severe symptoms. The total disease score was calculated by summing the 4 individual scores, with a maximum possible score of 12 per mouse.

### Statistics.

All statistical analyses were performed using GraphPad Prism (version 10.4.2, GraphPad Software). Two-tailed, unpaired or paired Student’s *t* tests and 1-way ANOVA were used as appropriate. *P* values of less than 0.05 were considered statistically significant. Data are presented as the mean ± SD.

### Study approval.

All animal procedures were approved by the IACUC of UCSD (protocol S09074).

### Data availability.

All data supporting the findings of this study are available within the article and its supplemental materials, including the Supporting Data Values file. The genomic data presented here are available in the NCBI’s GEO database (GEO GSE307681).

## Author contributions

T Numata was responsible for study conceptualization, experiments, formal analysis, visualization, and writing, reviewing, and editing the manuscript. MS, YN, FL, HC, JS, AAJ, MPI, and HL contributed to experiments and data curation. T Nakatsuji and KJC contributed to study methodology and data analysis. FL and HC also helped with animal experiments. HC, JS, and KJC contributed to bioinformatics and transcriptomics analysis. RLG supervised the study, handled project administration and funding acquisition, and reviewed and edited the manuscript.

## Funding support

This work is the result of NIH funding, in whole or in part, and is subject to the NIH Public Access Policy. Through acceptance of this federal funding, the NIH has been given a right to make the work publicly available in PubMed Central.

- NIH grants U01AI152038, R37AI052453, and P50AR080594 (to RLG).
- San Diego Center for AIDS Research (CFAR) (P30 AI036214).

## Supplementary Material

Supplemental data

Supporting data values

## Version 1 01/22/2026

In-Press Preview

## Version 2 03/02/2026

Electronic publication

## References

[1] Langan SM (2020). Atopic dermatitis. Lancet.

[2] Gandhi NA (2016). Targeting key proximal drivers of type 2 inflammation in disease. Nat Rev Drug Discov.

[3] Bieber T (2022). Atopic dermatitis: an expanding therapeutic pipeline for a complex disease. Nat Rev Drug Discov.

[4] Ogulur I (2025). Type 2 immunity in allergic diseases. Cell Mol Immunol.

[5] Kim BS (2013). TSLP elicits IL-33-independent innate lymphoid cell responses to promote skin inflammation. Sci Transl Med.

[6] Leyva-Castillo JM (2020). ILC2 activation by keratinocyte-derived IL-25 drives IL-13 production at sites of allergic skin inflammation. J Allergy Clin Immunol.

[7] Simpson EL (2016). Two phase 3 trials of dupilumab versus placebo in atopic dermatitis. N Engl J Med.

[8] Reich K (2022). Efficacy and safety of abrocitinib versus dupilumab in adults with moderate-to-severe atopic dermatitis: a randomised, double-blind, multicentre phase 3 trial. Lancet.

[9] Silverberg JI (2023). Two phase 3 trials of lebrikizumab for moderate-to-severe atopic dermatitis. N Engl J Med.

[10] Simpson EL (2022). Safety of tralokinumab in adult patients with moderate-to-severe atopic dermatitis: pooled analysis of five randomized, double-blind, placebo-controlled phase II and phase III trials. Br J Dermatol.

[11] Paller AS (2023). Efficacy and safety of tralokinumab in adolescents with moderate to severe atopic dermatitis: the phase 3 ECZTRA 6 randomized clinical trial. JAMA Dermatol.

[12] Sorrell JM, Caplan AI (2004). Fibroblast heterogeneity: more than skin deep. J Cell Sci.

[13] Francis L (2024). Inflammatory memory in psoriasis: From remission to recurrence. J Allergy Clin Immunol.

[14] Abbasi S (2020). Distinct regulatory programs control the latent regenerative potential of dermal fibroblasts during wound healing. Cell Stem Cell.

[15] O’Neill AM (2022). Antimicrobial production by perifollicular dermal preadipocytes is essential to the pathophysiology of acne. Sci Transl Med.

[16] Cavagnero KJ (2024). CXCL12^+^ dermal fibroblasts promote neutrophil recruitment and host defense by recognition of IL-17. J Exp Med.

[17] Jiao D (2016). NOD2 and TLR2 ligands trigger the activation of basophils and eosinophils by interacting with dermal fibroblasts in atopic dermatitis-like skin inflammation. Cell Mol Immunol.

[18] Bernard JJ (2012). Ultraviolet radiation damages self noncoding RNA and is detected by TLR3. Nat Med.

[19] Nakatsuji T (2017). Antimicrobials from human skin commensal bacteria protect against *Staphylococcus aureus* and are deficient in atopic dermatitis. Sci Transl Med.

[20] Nakatsuji T (2023). Competition between skin antimicrobial peptides and commensal bacteria in type 2 inflammation enables survival of S. aureus. Cell Rep.

[21] Eichenfield LF (2019). Infections in dupilumab clinical trials in atopic dermatitis: a comprehensive pooled analysis. Am J Clin Dermatol.

[22] Lavazais S (2023). IRAK4 inhibition dampens pathogenic processes driving inflammatory skin diseases. Sci Transl Med.

[23] Cavagnero KJ, Gallo RL (2022). Essential immune functions of fibroblasts in innate host defense. Front Immunol.

[24] Cavagnero KJ (2025). Positionally distinct interferon stimulated dermal immune acting fibroblasts promote neutrophil recruitment in Sweet syndrome. J Allergy Clin Immunol.

[25] He H (2020). Single-cell transcriptome analysis of human skin identifies novel fibroblast subpopulation and enrichment of immune subsets in atopic dermatitis. J Allergy Clin Immunol.

[26] Weidinger S (2018). Atopic dermatitis. Nat Rev Dis Primers.

[27] Choi YE (2020). Effects of Tenascin C on the integrity of extracellular matrix and skin aging. Int J Mol Sci.

[28] Cai X (2023). Tenascin C^+^ papillary fibroblasts facilitate neuro-immune interaction in a mouse model of psoriasis. Nat Commun.

[29] Shimba S (2004). EPAS1 promotes adipose differentiation in 3T3-L1 cells. J Biol Chem.

[30] Zhang H (2015). LGL1 modulates proliferation, apoptosis, and migration of human fetal lung fibroblasts. Am J Physiol Lung Cell Mol Physiol.

[31] Rudnik M (2021). Elevated fibronectin levels in profibrotic CD14^+^ monocytes and CD14^+^ macrophages in systemic sclerosis. Front Immunol.

[32] Zdzalik-Bielecka D (2021). The GAS6-AXL signaling pathway triggers actin remodeling that drives membrane ruffling, macropinocytosis, and cancer-cell invasion. Proc Natl Acad Sci U S A.

[33] Ko KI (2022). NF-κB perturbation reveals unique immunomodulatory functions in Prx1^+^ fibroblasts that promote development of atopic dermatitis. Sci Transl Med.

[34] Mizoguchi F (2018). Functionally distinct disease-associated fibroblast subsets in rheumatoid arthritis. Nat Commun.

[35] Shi Z (2024). The role of dermal fibroblasts in autoimmune skin diseases. Front Immunol.

[36] Nutan FN (2012). The effect of topically applied corticosteroids on interleukin 1β levels in patients with atopic dermatitis. J Eur Acad Dermatol Venereol.

[37] Schwartz C (2019). Spontaneous atopic dermatitis in mice with a defective skin barrier is independent of ILC2 and mediated by IL-1β. Allergy.

[38] Clement V (2022). Tridimensional cell culture of dermal fibroblasts promotes exosome-mediated secretion of extracellular matrix proteins. Sci Rep.

[39] Nakatsuji T (2016). Staphylococcus aureus exploits epidermal barrier defects in atopic dermatitis to trigger cytokine expression. J Invest Dermatol.

[40] Svensson V (2017). Power analysis of single-cell RNA-sequencing experiments. Nat Methods.

[41] Sallusto F (1997). Selective expression of the eotaxin receptor CCR3 by human T helper 2 cells. Science.

[42] Jinquan T (1999). Eotaxin activates T cells to chemotaxis and adhesion only if induced to express CCR3 by IL-2 together with IL-4. J Immunol.

[43] Heng TS (2008). The Immunological Genome Project: networks of gene expression in immune cells. Nat Immunol.

[44] Gebhardt T (2011). Different patterns of peripheral migration by memory CD4^+^ and CD8^+^ T cells. Nature.

[45] Grozdanovic M (2019). Novel peptide nanoparticle-biased antagonist of CCR3 blocks eosinophil recruitment and airway hyperresponsiveness. J Allergy Clin Immunol.

[46] Ochi H (1999). T helper cell type 2 cytokine-mediated comitogenic responses and CCR3 expression during differentiation of human mast cells in vitro. J Exp Med.

[47] Collington SJ (2010). The function of CCR3 on mouse bone marrow-derived mast cells in vitro. Immunology.

[48] Uguccioni M (1997). High expression of the chemokine receptor CCR3 in human blood basophils. Role in activation by eotaxin, MCP-4, and other chemokines. J Clin Invest.

[49] Hausmann OV (2011). Robust expression of CCR3 as a single basophil selection marker in flow cytometry. Allergy.

[50] Zhang W (2025). Basophil-VAMP7 is a vital regulator of skin barrier integrity and chronic itch. J Allergy Clin Immunol.

[51] Beaulieu S (2002). Expression of a functional eotaxin (CC chemokine ligand 11) receptor CCR3 by human dendritic cells. J Immunol.

[52] Puxeddu I (2006). The CC chemokine eotaxin/CCL11 has a selective profibrogenic effect on human lung fibroblasts. J Allergy Clin Immunol.

[53] Gaspar K (2013). The chemokine receptor CCR3 participates in tissue remodeling during atopic skin inflammation. J Dermatol Sci.

[54] Wakabayashi K (2021). Eotaxin-1/CCL11 is involved in cell migration in rheumatoid arthritis. Sci Rep.

[55] Struyf S (2009). Synergistic up-regulation of MCP-2/CCL8 activity is counteracted by chemokine cleavage, limiting its inflammatory and anti-tumoral effects. Eur J Immunol.

[56] Ge B (2017). Functional expression of CCL8 and its interaction with chemokine receptor CCR3. BMC Immunol.

[57] Rossi D, Zlotnik A (2000). The biology of chemokines and their receptors. Annu Rev Immunol.

[58] Heath H (1997). Chemokine receptor usage by human eosinophils. The importance of CCR3 demonstrated using an antagonistic monoclonal antibody. J Clin Invest.

[59] Stellato C (1997). Production of the novel C-C chemokine MCP-4 by airway cells and comparison of its biological activity to other C-C chemokines. J Clin Invest.

[60] Gupta S (2008). n-Nonanoyl-CCL14 (NNY-CCL14), a novel inhibitor of allergic airway inflammation is a partial agonist of human CCR2. Allergy.

[61] Forssmann U (1997). Eotaxin-2, a novel CC chemokine that is selective for the chemokine receptor CCR3, and acts like eotaxin on human eosinophil and basophil leukocytes. J Exp Med.

[62] Castan L (2017). Chemokine receptors in allergic diseases. Allergy.

[63] Boothby IC (2021). Early-life inflammation primes a T helper 2 cell-fibroblast niche in skin. Nature.

[64] Kim M (2022). Single-cell RNA-seq of primary bone marrow neutrophils from female and male adult mice. Sci Data.

[65] Lu S (2025). Transcriptome size matters for single-cell RNA-seq normalization and bulk deconvolution. Nat Commun.

[66] Guerrero-Juarez CF (2019). Single-cell analysis reveals fibroblast heterogeneity and myeloid-derived adipocyte progenitors in murine skin wounds. Nat Commun.

[67] Nakatsuji T (2021). Development of a human skin commensal microbe for bacteriotherapy of atopic dermatitis and use in a phase 1 randomized clinical trial. Nat Med.

[68] Mori A (2007). Selective suppression of Th2-mediated airway eosinophil infiltration by low-molecular weight CCR3 antagonists. Int Immunol.

[69] Lopez-Leal F (2024). Blockade of the CCR3 receptor reduces neutrophil recruitment to the lung during acute inflammation. J Leukoc Biol.

[70] Korsunsky I (2019). Fast, sensitive and accurate integration of single-cell data with Harmony. Nat Methods.

[71] Jin S (2021). Inference and analysis of cell-cell communication using CellChat. Nat Commun.

