# Supplementary material for: Dermal fibroblasts respond to IL-4 and IL-13 and promote T cell recruitment in atopic dermatitis
Source: J Clin Invest. 2026 Jan 22;136(5):e196108. doi: 10.1172/JCI196108 (PMC12948424; doi:10.1172/JCI196108)
Supplement: Supplemental data [file jci-136-196108-s054.pdf]

Figure S1

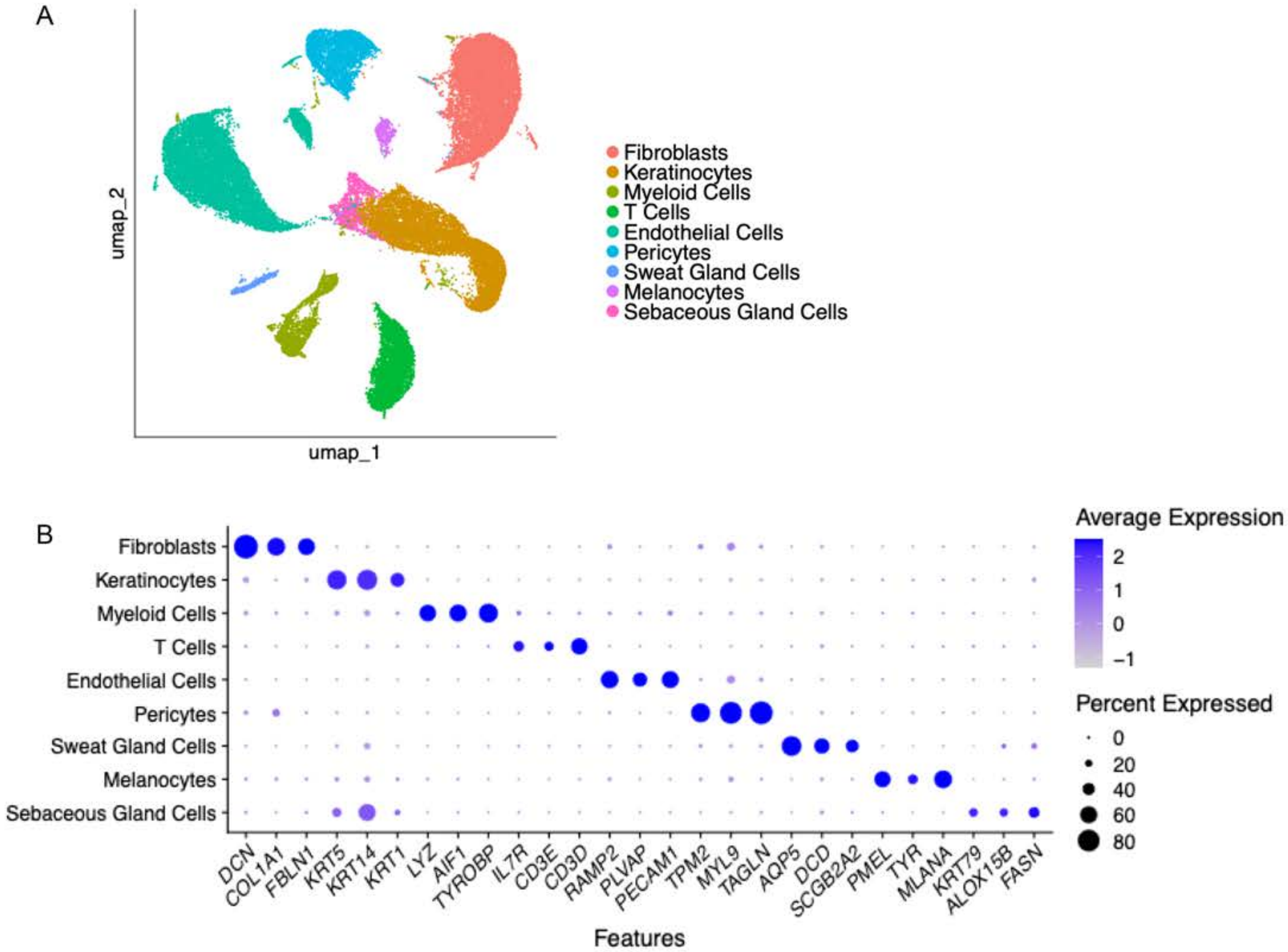
A
umap_2
umap_1
Fibroblasts
Keratinocytes
Myeloid Cells
T Cells
Endothelial Cells
Pericytes
Sweat Gland Cells
Melanocytes
Sebaceous Gland Cells
B
Fibroblasts
Keratinocytes
Myeloid Cells
T Cells
Endothelial Cells
Pericytes
Sweat Gland Cells
Melanocytes
Sebaceous Gland Cells
DCN
COL1A1
FBLN1
KRT5
KRT14
KRT1
LYZ
AIF1
TYROBP
IL7R
CD3E
CD3D
RAMP2
PLVAP
PECAM1
TPM2
MYL9
TAGLN
AQP5
DCD
SCGB2A2
PMEL
TYR
MLANA
KRT79
ALOX15B
FASN
Features
Average Expression
2
1
0
−1
Percent Expressed
0
20
40
60
80

Figure S1

C

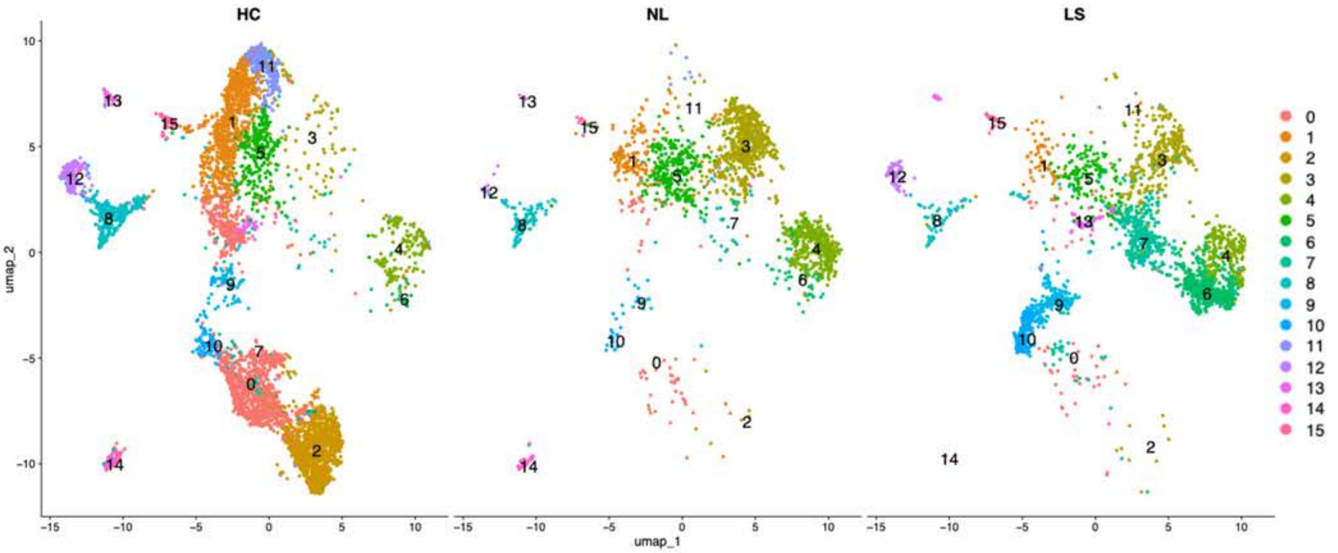

D

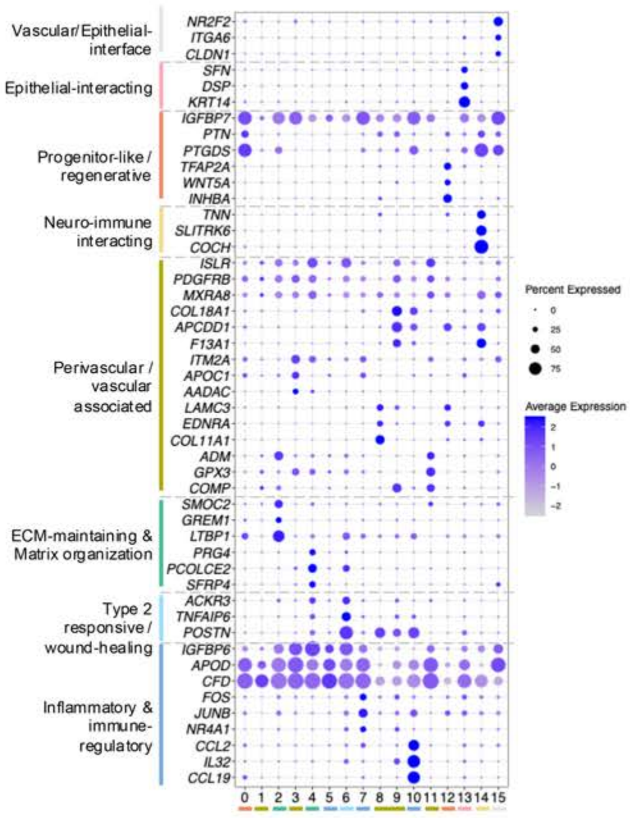

E

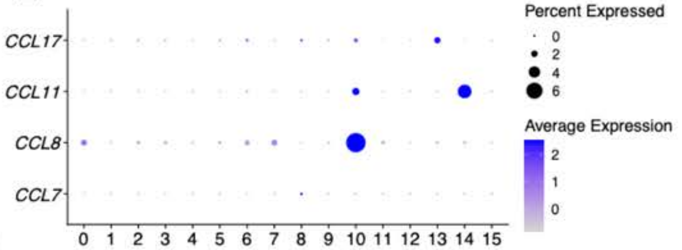

Figure S1

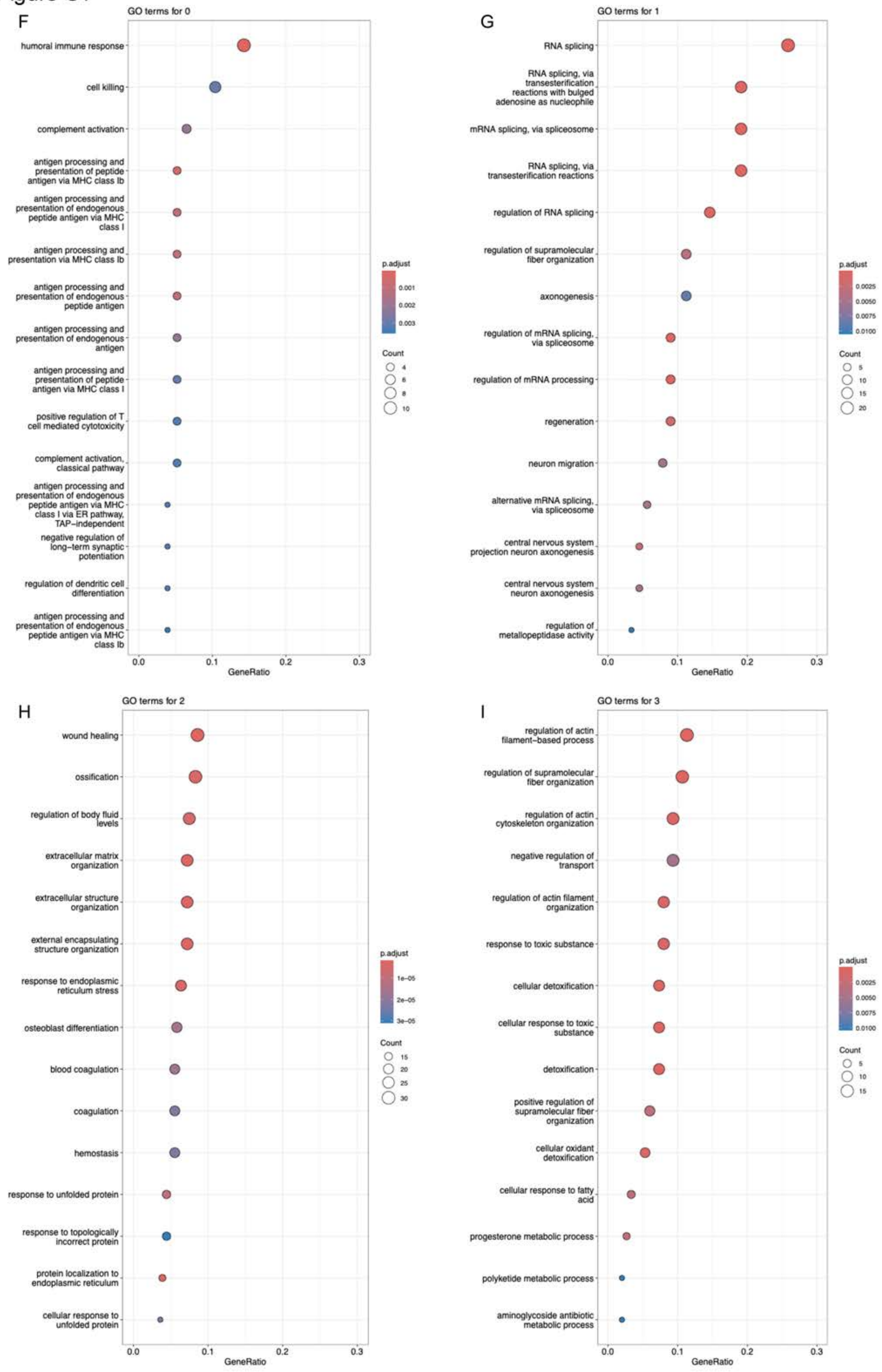
F
GO terms for 0
humoral immune response
cell killing
complement activation
antigen processing and presentation of peptide antigen via MHC class Ib
antigen processing and presentation of endogenous peptide antigen via MHC class I
antigen processing and presentation via MHC class Ib
antigen processing and presentation of endogenous peptide antigen
antigen processing and presentation of endogenous antigen
antigen processing and presentation of peptide antigen via MHC class I
positive regulation of T cell mediated cytotoxicity
complement activation, classical pathway
antigen processing and presentation of endogenous peptide antigen via MHC class I via ER pathway, TAP−independent
negative regulation of long−term synaptic potentiation
regulation of dendritic cell differentiation
antigen processing and presentation of endogenous peptide antigen via MHC class Ib
GeneRatio
p.adjust
Count
G
GO terms for 1
RNA splicing
RNA splicing, via transesterification reactions with bulged adenosine as nucleophile
mRNA splicing, via spliceosome
RNA splicing, via transesterification reactions
regulation of RNA splicing
regulation of supramolecular fiber organization
axonogenesis
regulation of mRNA splicing, via spliceosome
regulation of mRNA processing
regeneration
neuron migration
alternative mRNA splicing, via spliceosome
central nervous system projection neuron axonogenesis
central nervous system neuron axonogenesis
regulation of metallopeptidase activity
GeneRatio
p.adjust
Count
H
GO terms for 2
wound healing
ossification
regulation of body fluid levels
extracellular matrix organization
extracellular structure organization
external encapsulating structure organization
response to endoplasmic reticulum stress
osteoblast differentiation
blood coagulation
coagulation
hemostasis
response to unfolded protein
response to topologically incorrect protein
protein localization to endoplasmic reticulum
cellular response to unfolded protein
GeneRatio
p.adjust
Count
I
GO terms for 3
regulation of actin filament−based process
regulation of supramolecular fiber organization
regulation of actin cytoskeleton organization
negative regulation of transport
regulation of actin filament organization
response to toxic substance
cellular detoxification
cellular response to toxic substance
detoxification
positive regulation of supramolecular fiber organization
cellular oxidant detoxification
cellular response to fatty acid
progesterone metabolic process
polyketide metabolic process
aminoglycoside antibiotic metabolic process
GeneRatio
p.adjust
Count

Figure S1

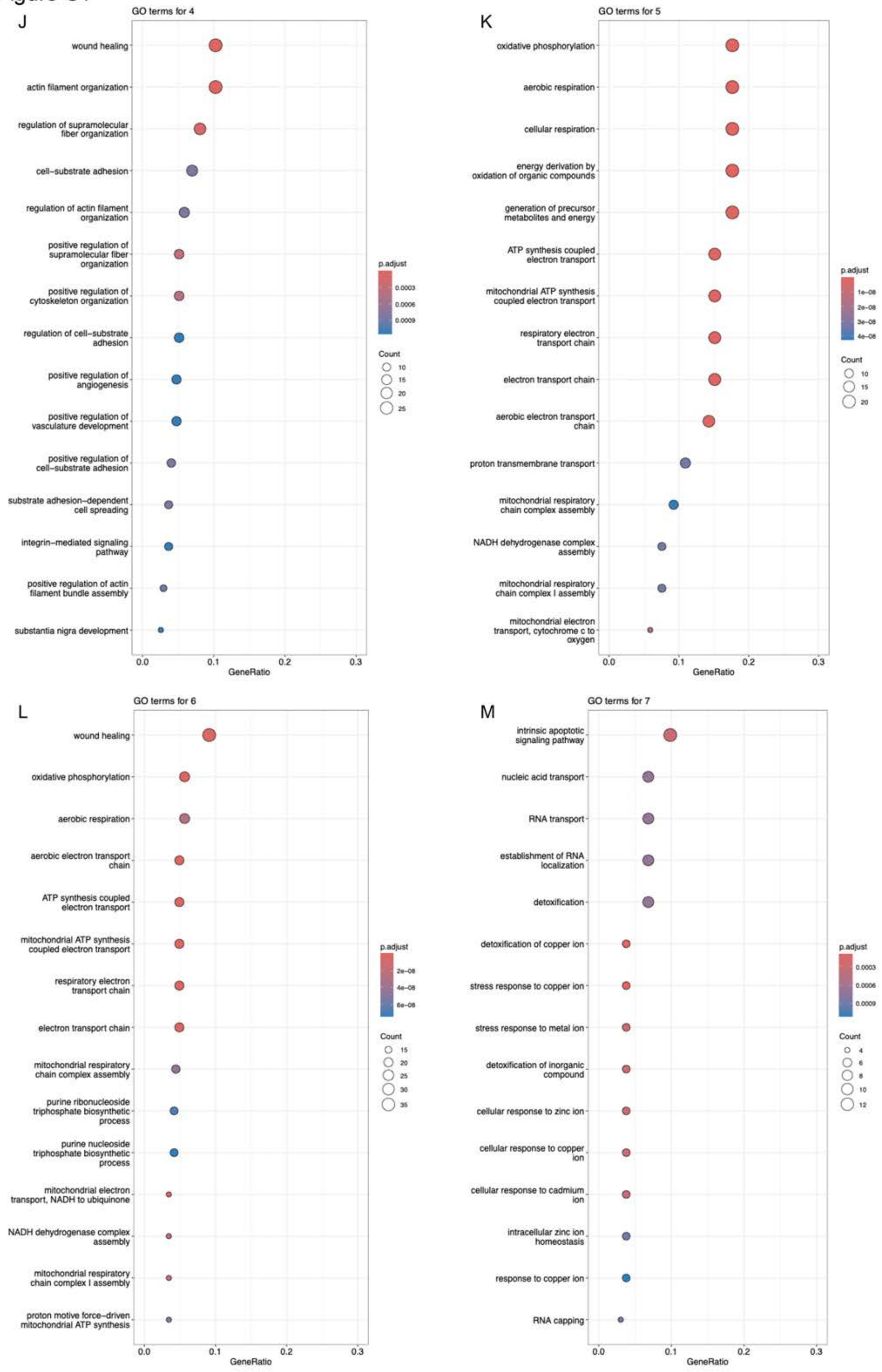
J
GO terms for 4
wound healing
actin filament organization
regulation of supramolecular fiber organization
cell−substrate adhesion
regulation of actin filament organization
positive regulation of supramolecular fiber organization
positive regulation of cytoskeleton organization
regulation of cell−substrate adhesion
positive regulation of angiogenesis
positive regulation of vasculature development
positive regulation of cell−substrate adhesion
substrate adhesion−dependent cell spreading
integrin−mediated signaling pathway
positive regulation of actin filament bundle assembly
substantia nigra development
0.0
0.1
0.2
0.3
GeneRatio
p.adjust
0.0003
0.0006
0.0009
Count
10
15
20
25
K
GO terms for 5
oxidative phosphorylation
aerobic respiration
cellular respiration
energy derivation by oxidation of organic compounds
generation of precursor metabolites and energy
ATP synthesis coupled electron transport
mitochondrial ATP synthesis coupled electron transport
respiratory electron transport chain
electron transport chain
aerobic electron transport chain
proton transmembrane transport
mitochondrial respiratory chain complex assembly
NADH dehydrogenase complex assembly
mitochondrial respiratory chain complex I assembly
mitochondrial electron transport, cytochrome c to oxygen
0.0
0.1
0.2
0.3
GeneRatio
p.adjust
1e−08
2e−08
3e−08
4e−08
Count
10
15
20
L
GO terms for 6
wound healing
oxidative phosphorylation
aerobic respiration
aerobic electron transport chain
ATP synthesis coupled electron transport
mitochondrial ATP synthesis coupled electron transport
respiratory electron transport chain
electron transport chain
mitochondrial respiratory chain complex assembly
purine ribonucleoside triphosphate biosynthetic process
purine nucleoside triphosphate biosynthetic process
mitochondrial electron transport, NADH to ubiquinone
NADH dehydrogenase complex assembly
mitochondrial respiratory chain complex I assembly
proton motive force−driven mitochondrial ATP synthesis
0.0
0.1
0.2
0.3
GeneRatio
p.adjust
2e−08
4e−08
6e−08
Count
15
20
25
30
35
M
GO terms for 7
intrinsic apoptotic signaling pathway
nucleic acid transport
RNA transport
establishment of RNA localization
detoxification
detoxification of copper ion
stress response to copper ion
stress response to metal ion
detoxification of inorganic compound
cellular response to zinc ion
cellular response to copper ion
cellular response to cadmium ion
intracellular zinc ion homeostasis
response to copper ion
RNA capping
0.0
0.1
0.2
0.3
GeneRatio
p.adjust
0.0003
0.0006
0.0009
Count
4
6
8
10
12

Figure S1

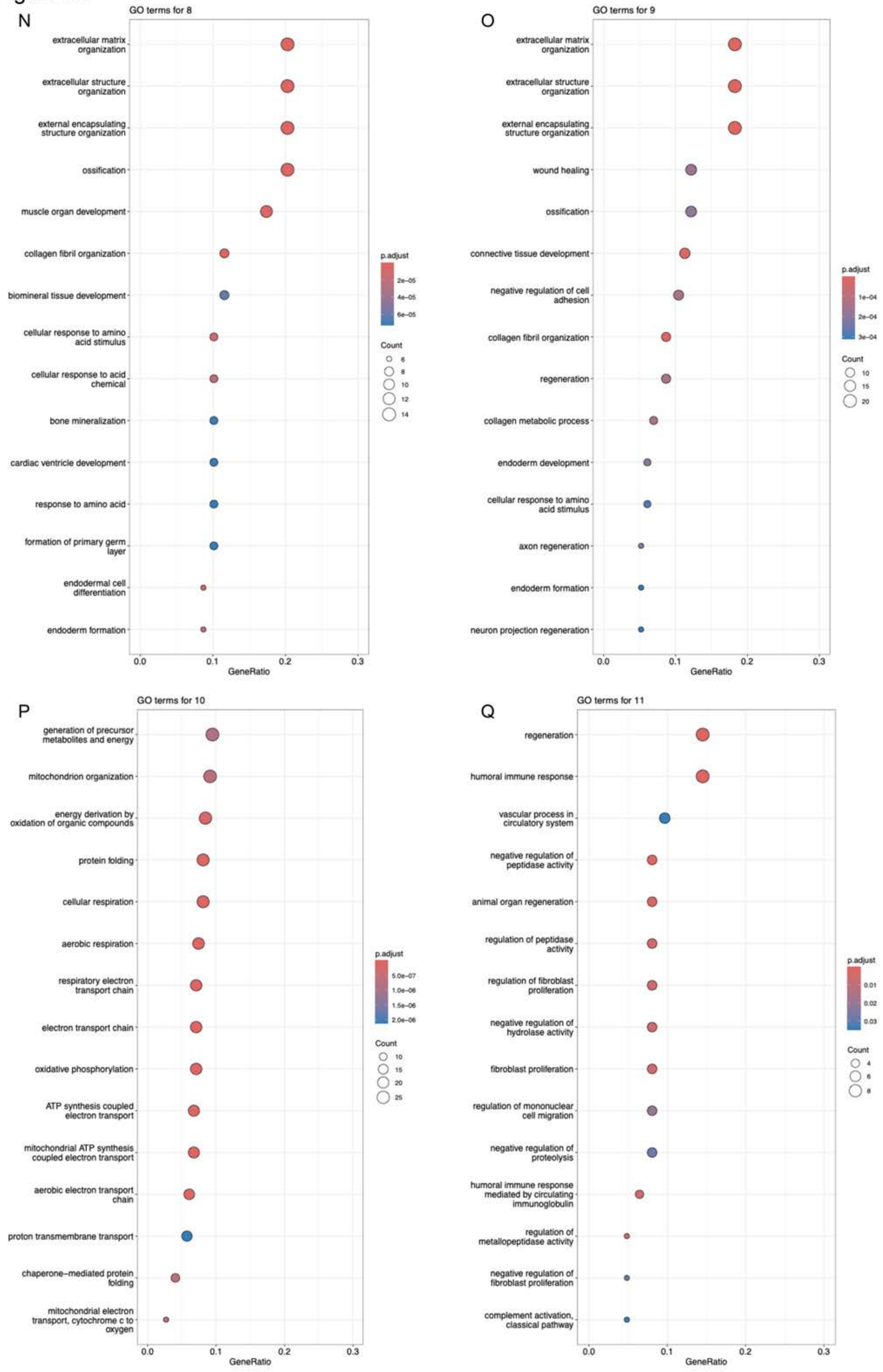
N
GO terms for 8
extracellular matrix organization
extracellular structure organization
external encapsulating structure organization
ossification
muscle organ development
collagen fibril organization
biomineral tissue development
cellular response to amino acid stimulus
cellular response to acid chemical
bone mineralization
cardiac ventricle development
response to amino acid
formation of primary germ layer
endodermal cell differentiation
endoderm formation
0.0 0.1 0.2 0.3
GeneRatio
p.adjust
2e-05
4e-05
6e-05
Count
6
8
10
12
14
O
GO terms for 9
extracellular matrix organization
extracellular structure organization
external encapsulating structure organization
wound healing
ossification
connective tissue development
negative regulation of cell adhesion
collagen fibril organization
regeneration
collagen metabolic process
endoderm development
cellular response to amino acid stimulus
axon regeneration
endoderm formation
neuron projection regeneration
0.0 0.1 0.2 0.3
GeneRatio
p.adjust
1e-04
2e-04
3e-04
Count
10
15
20
P
GO terms for 10
generation of precursor metabolites and energy
mitochondrion organization
energy derivation by oxidation of organic compounds
protein folding
cellular respiration
aerobic respiration
respiratory electron transport chain
electron transport chain
oxidative phosphorylation
ATP synthesis coupled electron transport
mitochondrial ATP synthesis coupled electron transport
aerobic electron transport chain
proton transmembrane transport
chaperone−mediated protein folding
mitochondrial electron transport, cytochrome c to oxygen
0.0 0.1 0.2 0.3
GeneRatio
p.adjust
5.0e−07
1.0e−06
1.5e−06
2.0e−06
Count
10
15
20
25
Q
GO terms for 11
regeneration
humoral immune response
vascular process in circulatory system
negative regulation of peptidase activity
animal organ regeneration
regulation of peptidase activity
regulation of fibroblast proliferation
negative regulation of hydrolase activity
fibroblast proliferation
regulation of mononuclear cell migration
negative regulation of proteolysis
humoral immune response mediated by circulating immunoglobulin
regulation of metallopeptidase activity
negative regulation of fibroblast proliferation
complement activation, classical pathway
0.0 0.1 0.2 0.3
GeneRatio
p.adjust
0.01
0.02
0.03
Count
4
6
8

Figure S1

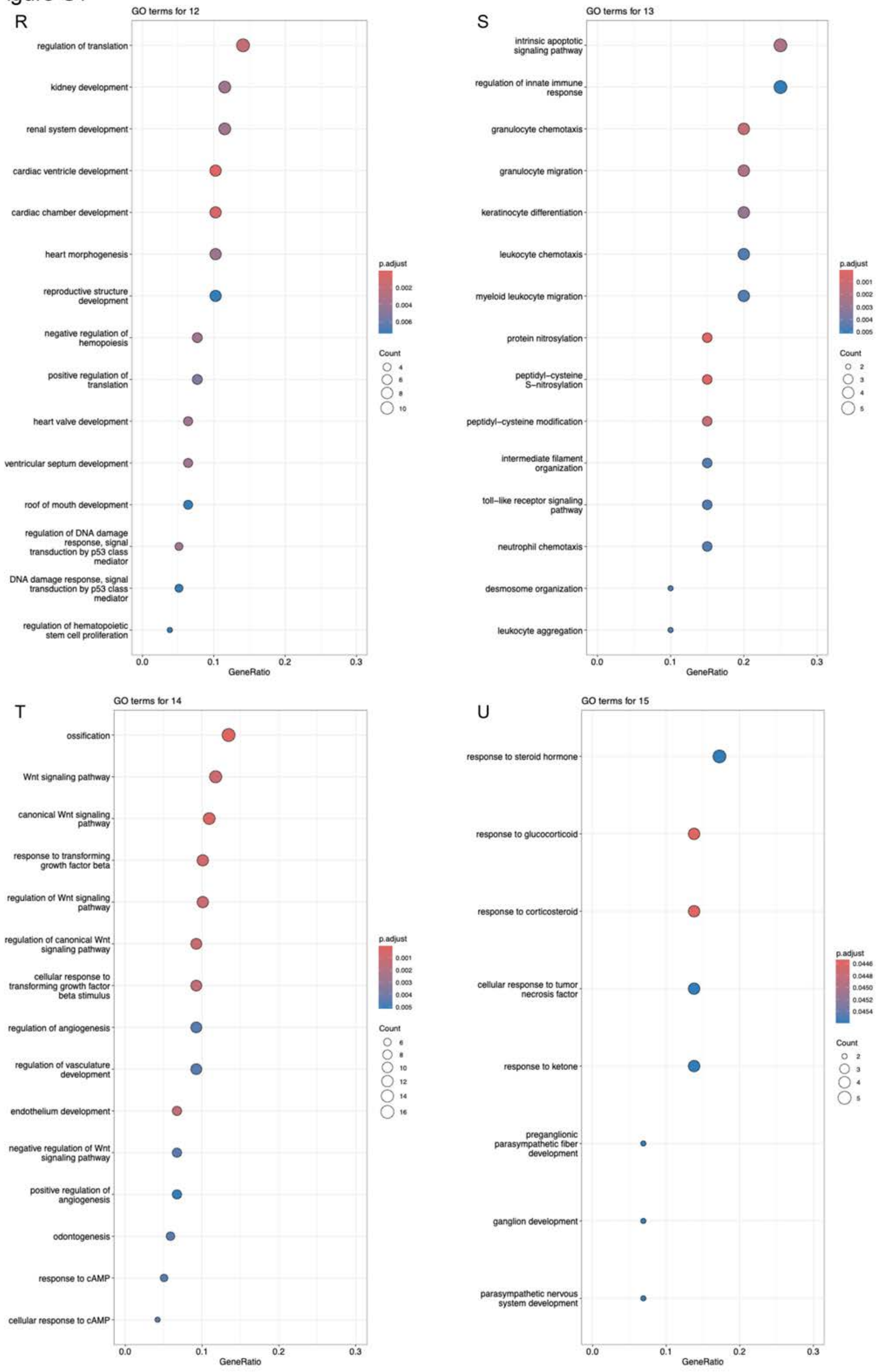
R
GO terms for 12
regulation of translation
kidney development
renal system development
cardiac ventricle development
cardiac chamber development
heart morphogenesis
reproductive structure development
negative regulation of hemopoiesis
positive regulation of translation
heart valve development
ventricular septum development
roof of mouth development
regulation of DNA damage response, signal transduction by p53 class mediator
DNA damage response, signal transduction by p53 class mediator
regulation of hematopoietic stem cell proliferation
0.0
0.1
0.2
0.3
GeneRatio
p.adjust
0.002
0.004
0.006
Count
4
6
8
10
S
GO terms for 13
intrinsic apoptotic signaling pathway
regulation of innate immune response
granulocyte chemotaxis
granulocyte migration
keratinocyte differentiation
leukocyte chemotaxis
myeloid leukocyte migration
protein nitrosylation
peptidyl−cysteine S−nitrosylation
peptidyl−cysteine modification
intermediate filament organization
toll−like receptor signaling pathway
neutrophil chemotaxis
desmosome organization
leukocyte aggregation
0.0
0.1
0.2
0.3
GeneRatio
p.adjust
0.001
0.002
0.003
0.004
0.005
Count
2
3
4
5
T
GO terms for 14
ossification
Wnt signaling pathway
canonical Wnt signaling pathway
response to transforming growth factor beta
regulation of Wnt signaling pathway
regulation of canonical Wnt signaling pathway
cellular response to transforming growth factor beta stimulus
regulation of angiogenesis
regulation of vasculature development
endothelium development
negative regulation of Wnt signaling pathway
positive regulation of angiogenesis
odontogenesis
response to cAMP
cellular response to cAMP
0.0
0.1
0.2
0.3
GeneRatio
p.adjust
0.001
0.002
0.003
0.004
0.005
Count
6
8
10
12
14
16
U
GO terms for 15
response to steroid hormone
response to glucocorticoid
response to corticosteroid
cellular response to tumor necrosis factor
response to ketone
preganglionic parasympathetic fiber development
ganglion development
parasympathetic nervous system development
0.0
0.1
0.2
0.3
GeneRatio
p.adjust
0.0446
0.0448
0.0450
0.0452
0.0454
Count
2
3
4
5

Figure S2

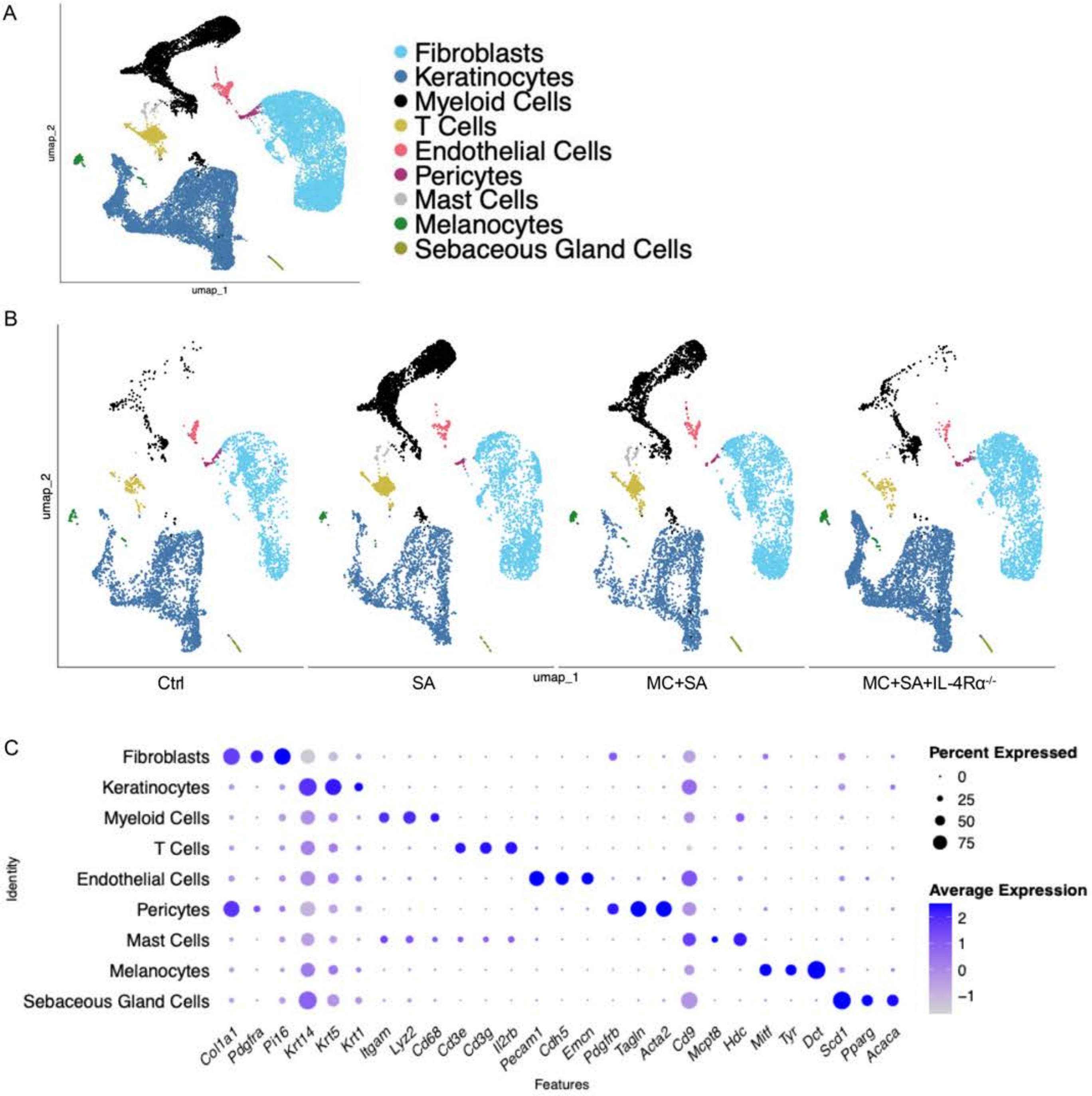
A
Fibroblasts
Keratinocytes
Myeloid Cells
T Cells
Endothelial Cells
Pericytes
Mast Cells
Melanocytes
Sebaceous Gland Cells
umap_1
umap_2
B
Ctrl
SA
MC+SA
MC+SA+IL-4Rα-/-
umap_1
umap_2
C
Percent Expressed
0
25
50
75
Average Expression
2
1
0
−1
Identity
Features
Col1a1
Pdgfra
Pi16
Krt14
Krt5
Krt1
Itgam
Lyz2
Cd68
Cd3e
Cd3g
Il2rb
Pecam1
Cdh5
Emcn
Pdgfrb
Tagln
Acta2
Cd9
Mcpt8
Hdc
Mitf
Tyr
Dct
Scd1
Pparg
Acaca

Figure S2

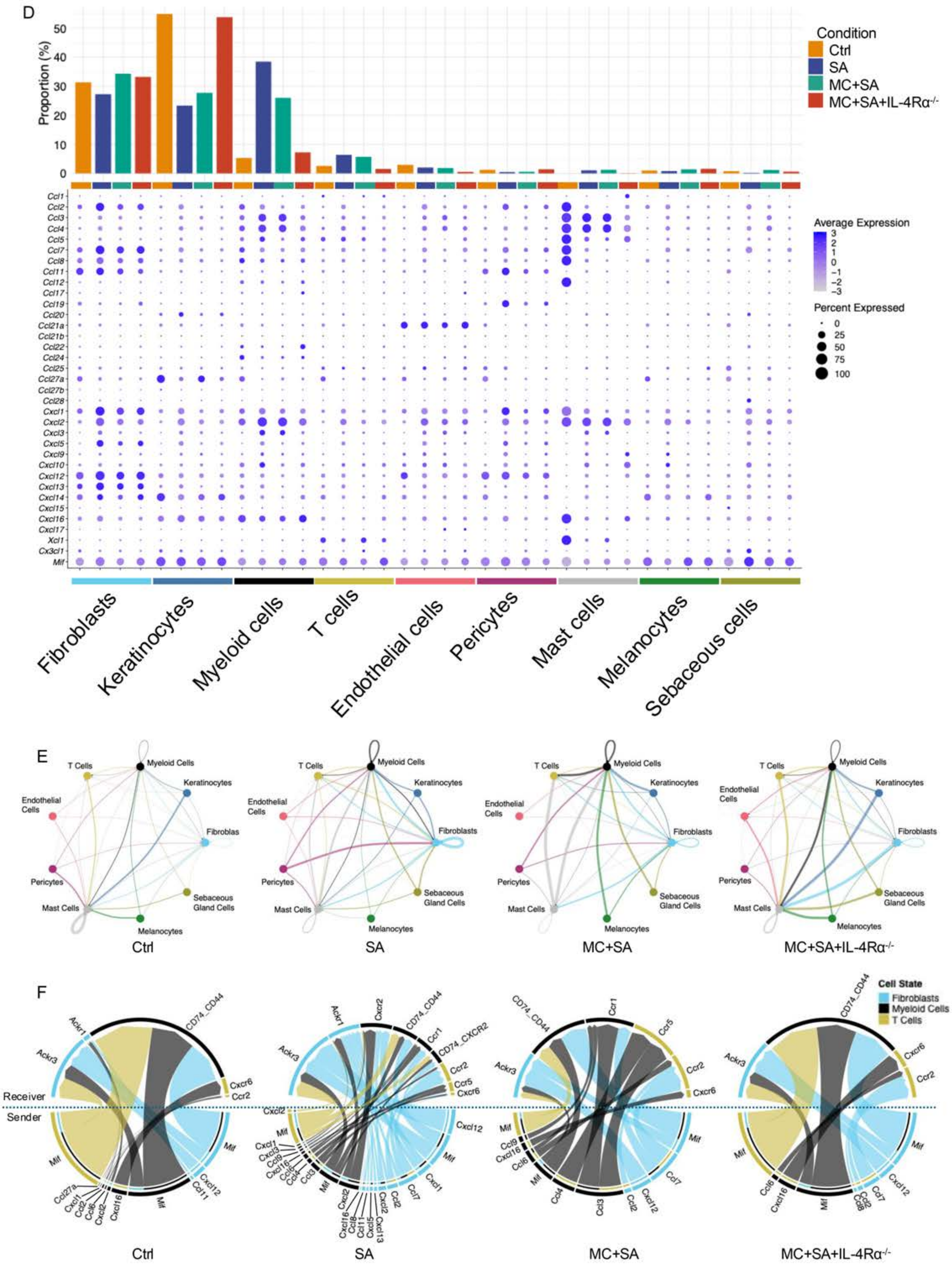
D
Proportion (%)
Condition
Ctrl
SA
MC+SA
MC+SA+IL-4Rα-/-
Average Expression
Percent Expressed
Ccl1
Ccl2
Ccl3
Ccl4
Ccl5
Ccl7
Ccl8
Ccl11
Ccl12
Ccl17
Ccl19
Ccl20
Ccl21a
Ccl21b
Ccl22
Ccl24
Ccl25
Ccl27a
Ccl27b
Ccl28
Cxcl1
Cxcl2
Cxcl3
Cxcl5
Cxcl9
Cxcl10
Cxcl12
Cxcl13
Cxcl14
Cxcl15
Cxcl16
Cxcl17
Xcl1
Cx3cl1
Mif
Fibroblasts
Keratinocytes
Myeloid cells
T cells
Endothelial cells
Pericytes
Mast cells
Melanocytes
Sebaceous cells
E
T Cells
Myeloid Cells
Keratinocytes
Endothelial Cells
Fibroblasts
Pericytes
Mast Cells
Sebaceous Gland Cells
Melanocytes
Ctrl
SA
MC+SA
MC+SA+IL-4Rα-/-
F
Cell State
Fibroblasts
Myeloid Cells
T Cells
Receiver
Sender
Ackr1
Ackr3
CD74_CD44
CD74_CXCR2
Cxcr2
Ccr1
Ccr2
Ccr5
Cxcr6
Mif
Cxcl12
Ccl11
Cxcl16
Ccl7
Ccl6
Ccl4
Ccl3
Ccl2
Ccl8
Ccl9
Cxcl2
Ctrl
SA
MC+SA
MC+SA+IL-4Rα-/-

Figure S2

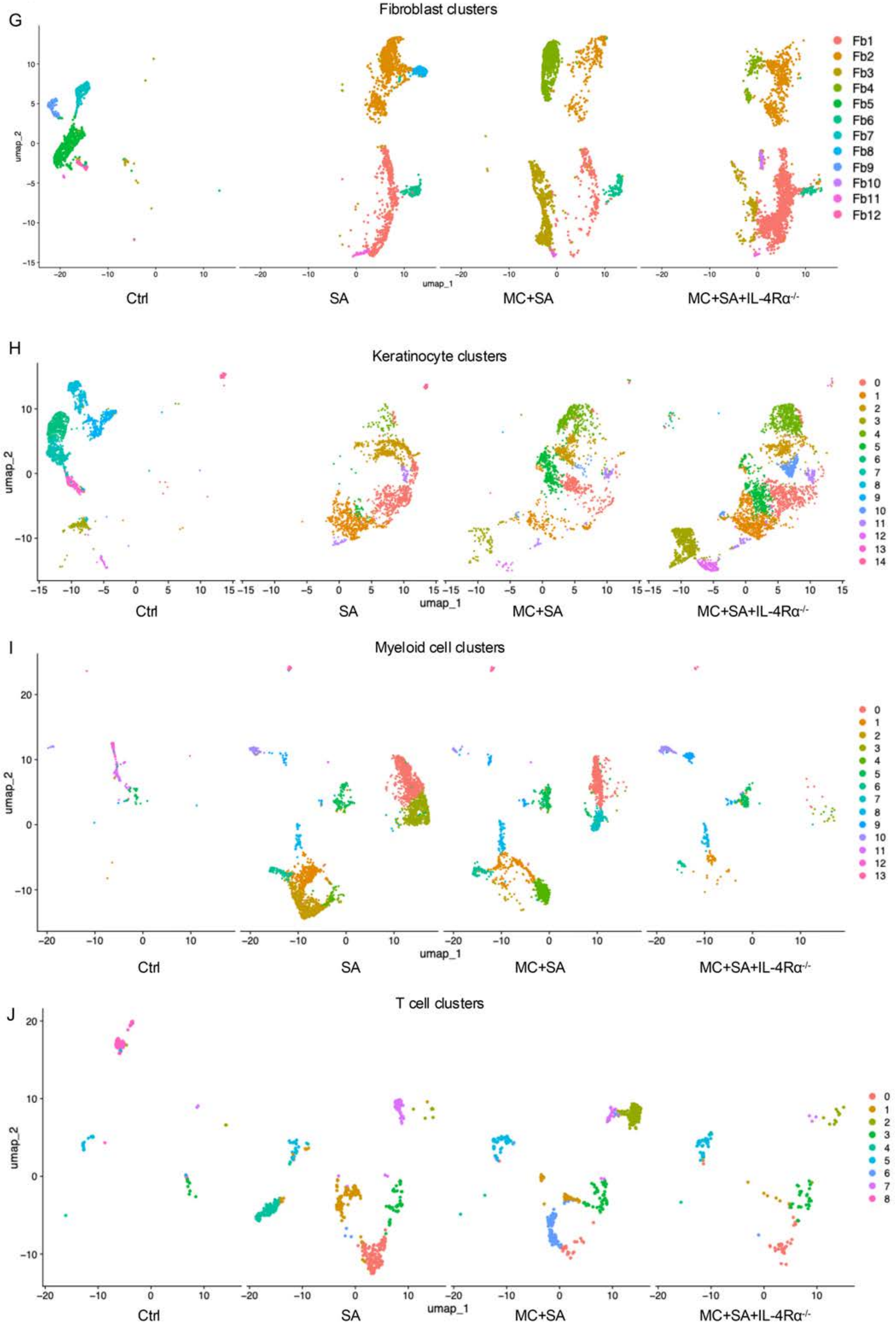

G
Fibroblast clusters
Fb1
Fb2
Fb3
Fb4
Fb5
Fb6
Fb7
Fb8
Fb9
Fb10
Fb11
Fb12
umap_1
umap_2
Ctrl
SA
MC+SA
MC+SA+IL-4Rα-/-
H
Keratinocyte clusters
0
1
2
3
4
5
6
7
8
9
10
11
12
13
14
umap_1
umap_2
Ctrl
SA
MC+SA
MC+SA+IL-4Rα-/-
I
Myeloid cell clusters
0
1
2
3
4
5
6
7
8
9
10
11
12
13
umap_1
umap_2
Ctrl
SA
MC+SA
MC+SA+IL-4Rα-/-
J
T cell clusters
0
1
2
3
4
5
6
7
8
umap_1
umap_2
Ctrl
SA
MC+SA
MC+SA+IL-4Rα-/-

Figure S2

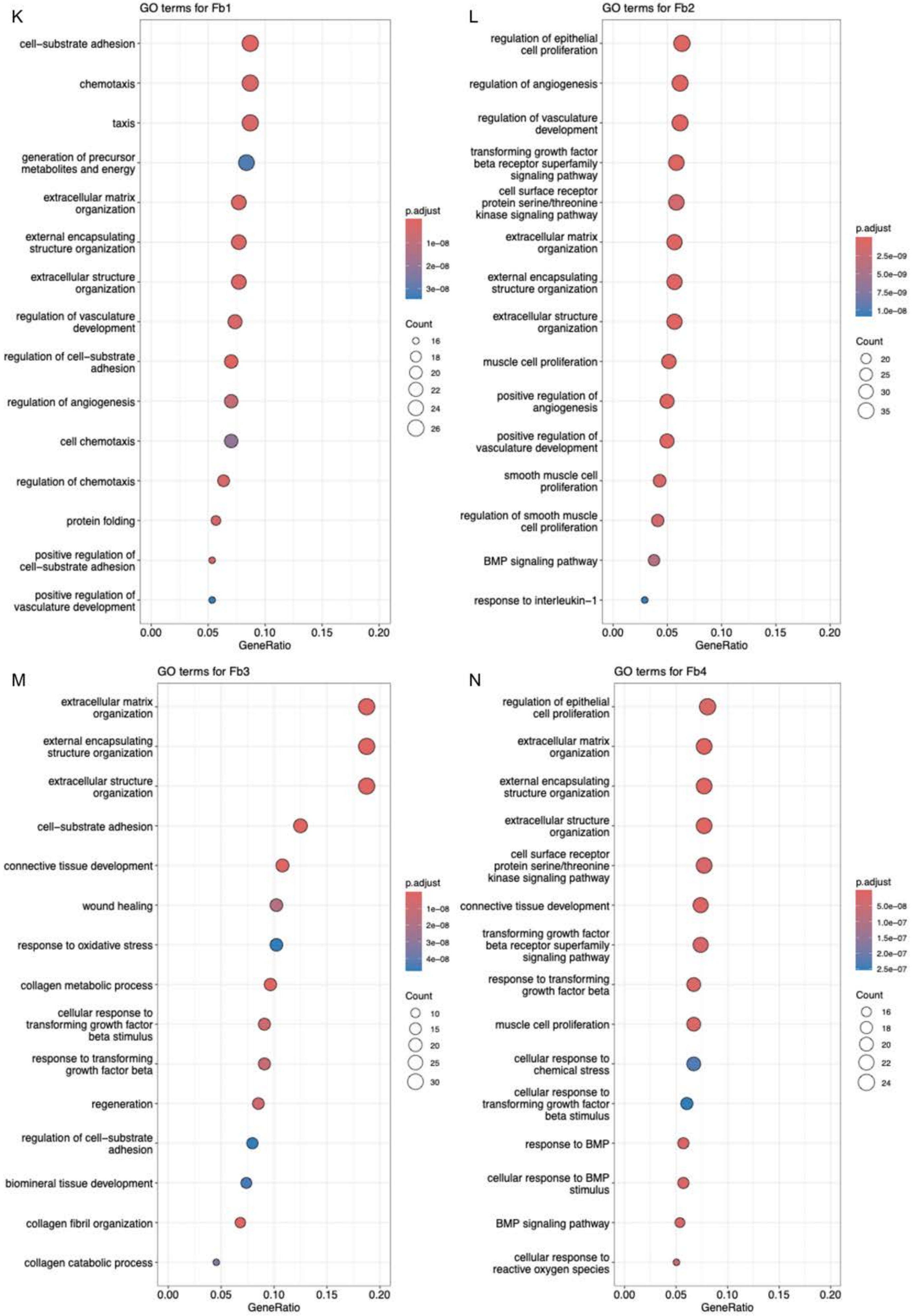
K
GO terms for Fb1
cell−substrate adhesion
chemotaxis
taxis
generation of precursor metabolites and energy
extracellular matrix organization
external encapsulating structure organization
extracellular structure organization
regulation of vasculature development
regulation of cell−substrate adhesion
regulation of angiogenesis
cell chemotaxis
regulation of chemotaxis
protein folding
positive regulation of cell−substrate adhesion
positive regulation of vasculature development
0.00 0.05 0.10 0.15 0.20
GeneRatio
p.adjust
1e−08
2e−08
3e−08
Count
16
18
20
22
24
26
L
GO terms for Fb2
regulation of epithelial cell proliferation
regulation of angiogenesis
regulation of vasculature development
transforming growth factor beta receptor superfamily signaling pathway
cell surface receptor protein serine/threonine kinase signaling pathway
extracellular matrix organization
external encapsulating structure organization
extracellular structure organization
muscle cell proliferation
positive regulation of angiogenesis
positive regulation of vasculature development
smooth muscle cell proliferation
regulation of smooth muscle cell proliferation
BMP signaling pathway
response to interleukin−1
0.00 0.05 0.10 0.15 0.20
GeneRatio
p.adjust
2.5e−09
5.0e−09
7.5e−09
1.0e−08
Count
20
25
30
35
M
GO terms for Fb3
extracellular matrix organization
external encapsulating structure organization
extracellular structure organization
cell−substrate adhesion
connective tissue development
wound healing
response to oxidative stress
collagen metabolic process
cellular response to transforming growth factor beta stimulus
response to transforming growth factor beta
regeneration
regulation of cell−substrate adhesion
biomineral tissue development
collagen fibril organization
collagen catabolic process
0.00 0.05 0.10 0.15 0.20
GeneRatio
p.adjust
1e−08
2e−08
3e−08
4e−08
Count
10
15
20
25
30
N
GO terms for Fb4
regulation of epithelial cell proliferation
extracellular matrix organization
external encapsulating structure organization
extracellular structure organization
cell surface receptor protein serine/threonine kinase signaling pathway
connective tissue development
transforming growth factor beta receptor superfamily signaling pathway
response to transforming growth factor beta
muscle cell proliferation
cellular response to chemical stress
cellular response to transforming growth factor beta stimulus
response to BMP
cellular response to BMP stimulus
BMP signaling pathway
cellular response to reactive oxygen species
0.00 0.05 0.10 0.15 0.20
GeneRatio
p.adjust
5.0e−08
1.0e−07
1.5e−07
2.0e−07
2.5e−07
Count
16
18
20
22
24

Figure S2

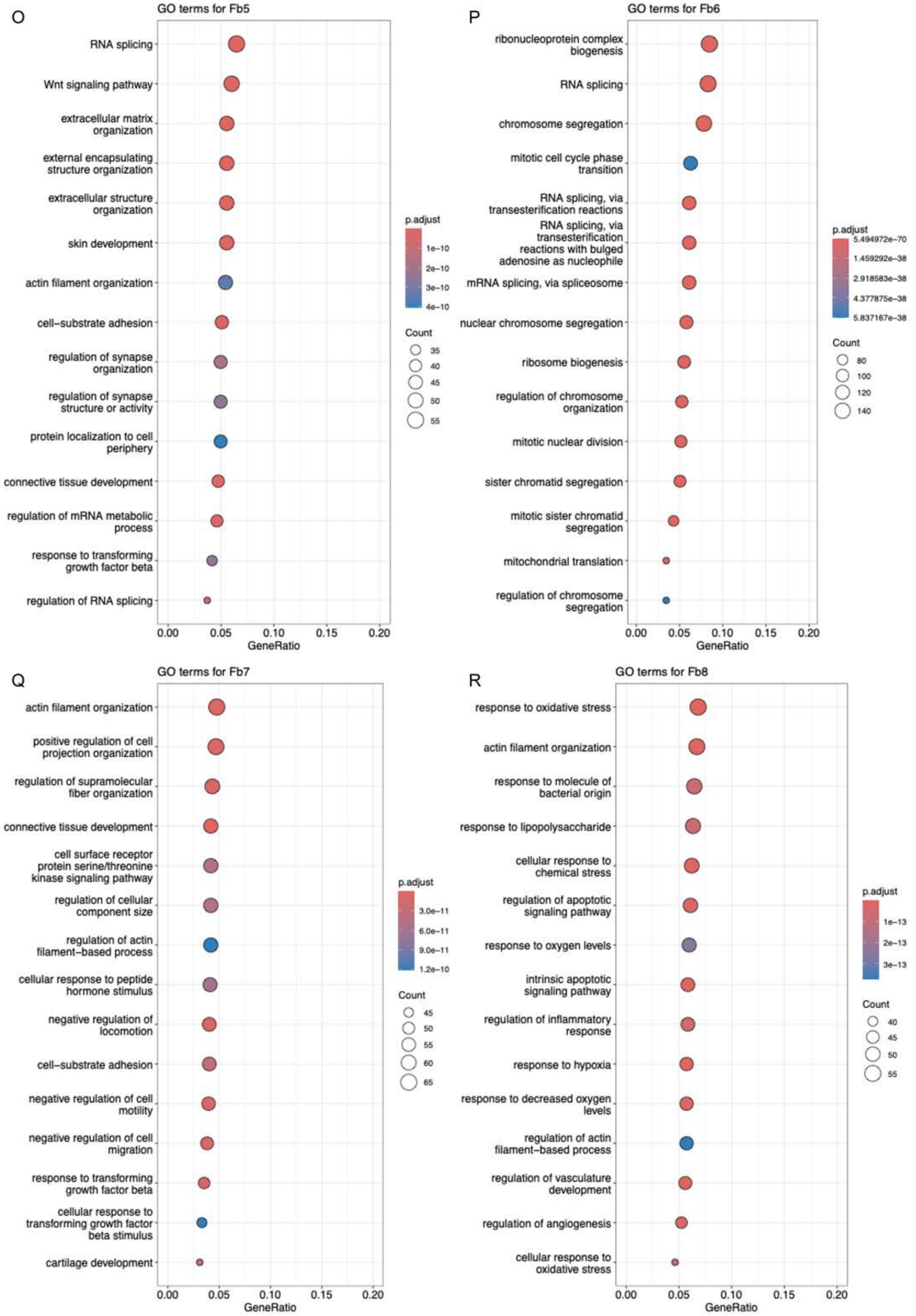

O
GO terms for Fb5
RNA splicing
Wnt signaling pathway
extracellular matrix organization
external encapsulating structure organization
extracellular structure organization
skin development
actin filament organization
cell−substrate adhesion
regulation of synapse organization
regulation of synapse structure or activity
protein localization to cell periphery
connective tissue development
regulation of mRNA metabolic process
response to transforming growth factor beta
regulation of RNA splicing
0.00 0.05 0.10 0.15 0.20
GeneRatio
p.adjust
1e−10
2e−10
3e−10
4e−10
Count
35
40
45
50
55
P
GO terms for Fb6
ribonucleoprotein complex biogenesis
RNA splicing
chromosome segregation
mitotic cell cycle phase transition
RNA splicing, via transesterification reactions
RNA splicing, via transesterification reactions with bulged adenosine as nucleophile
mRNA splicing, via spliceosome
nuclear chromosome segregation
ribosome biogenesis
regulation of chromosome organization
mitotic nuclear division
sister chromatid segregation
mitotic sister chromatid segregation
mitochondrial translation
regulation of chromosome segregation
0.00 0.05 0.10 0.15 0.20
GeneRatio
p.adjust
5.494972e−70
1.459292e−38
2.918583e−38
4.377875e−38
5.837167e−38
Count
80
100
120
140
Q
GO terms for Fb7
actin filament organization
positive regulation of cell projection organization
regulation of supramolecular fiber organization
connective tissue development
cell surface receptor protein serine/threonine kinase signaling pathway
regulation of cellular component size
regulation of actin filament−based process
cellular response to peptide hormone stimulus
negative regulation of locomotion
cell−substrate adhesion
negative regulation of cell motility
negative regulation of cell migration
response to transforming growth factor beta
cellular response to transforming growth factor beta stimulus
cartilage development
0.00 0.05 0.10 0.15 0.20
GeneRatio
p.adjust
3.0e−11
6.0e−11
9.0e−11
1.2e−10
Count
45
50
55
60
65
R
GO terms for Fb8
response to oxidative stress
actin filament organization
response to molecule of bacterial origin
response to lipopolysaccharide
cellular response to chemical stress
regulation of apoptotic signaling pathway
response to oxygen levels
intrinsic apoptotic signaling pathway
regulation of inflammatory response
response to hypoxia
response to decreased oxygen levels
regulation of actin filament−based process
regulation of vasculature development
regulation of angiogenesis
cellular response to oxidative stress
0.00 0.05 0.10 0.15 0.20
GeneRatio
p.adjust
1e−13
2e−13
3e−13
Count
40
45
50
55

Figure S2

S

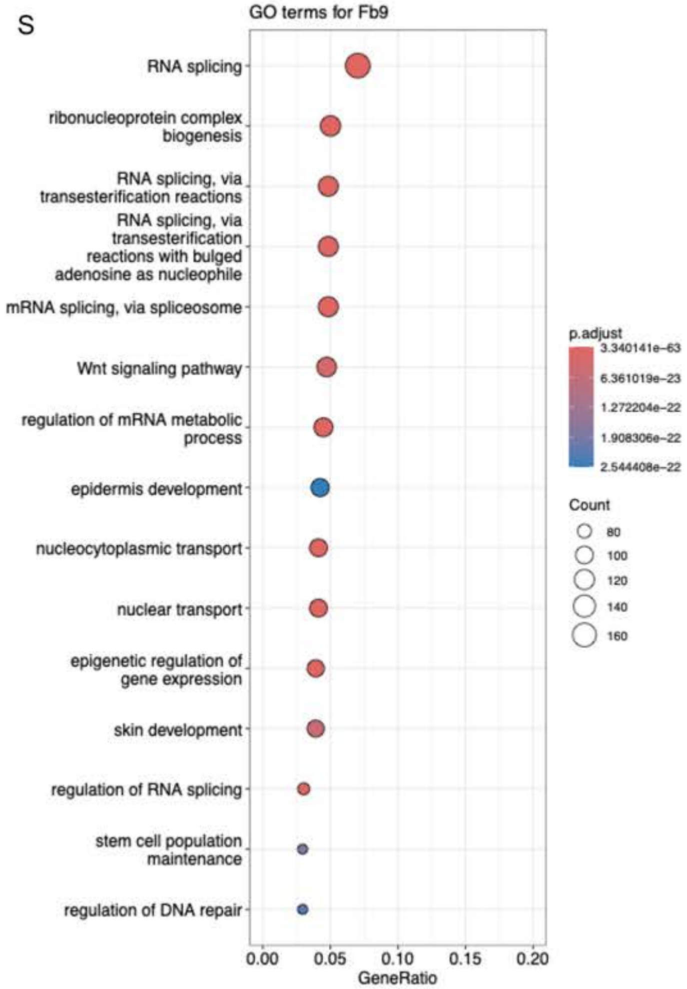

T

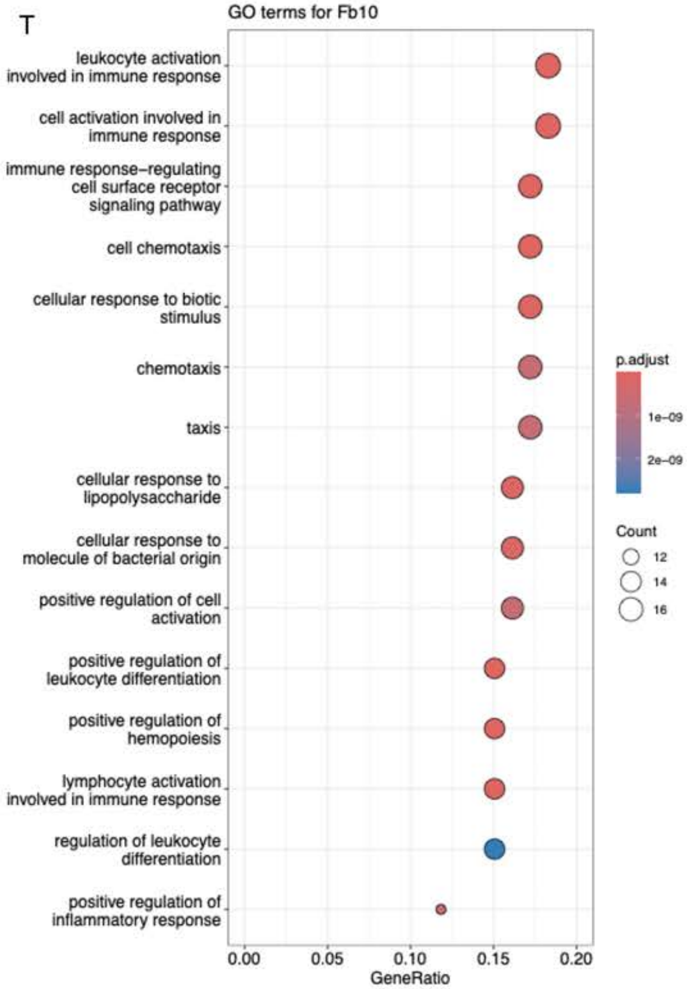

U

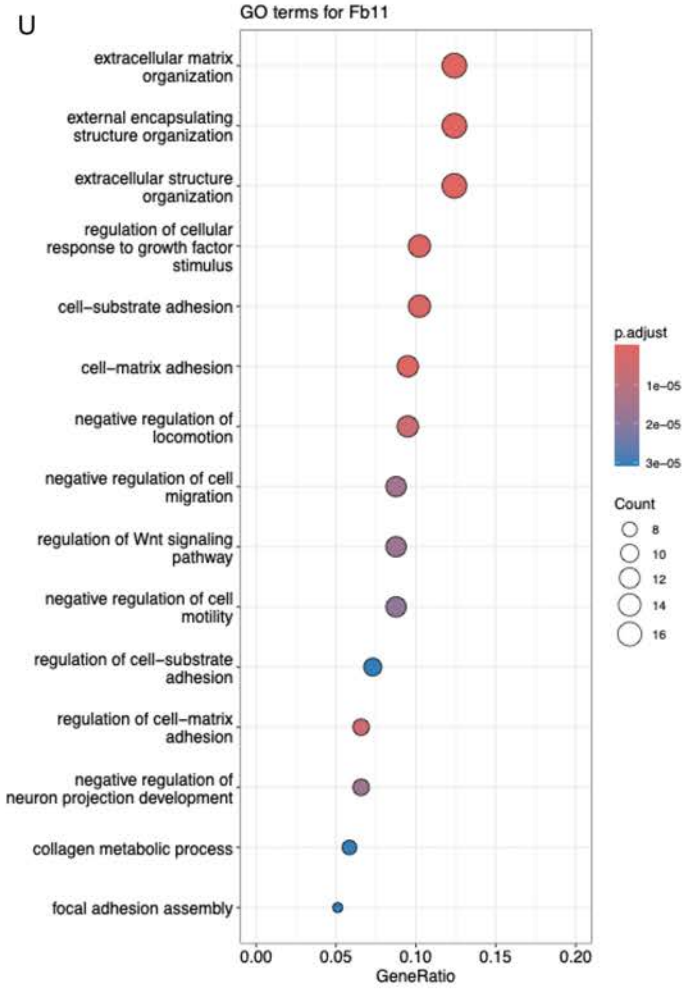

V

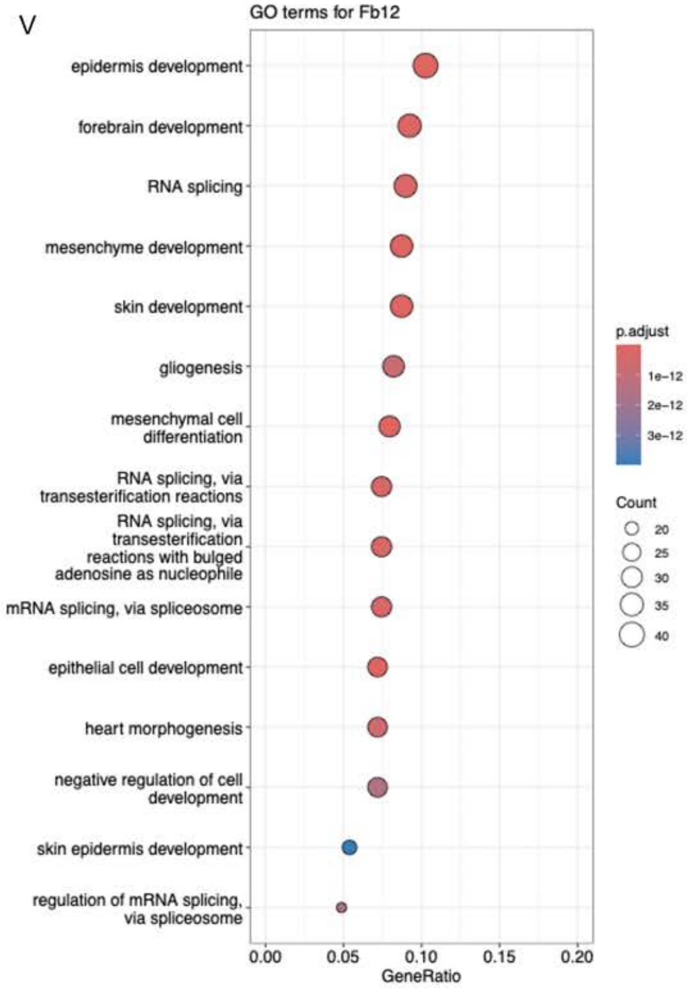

Figure S2

W

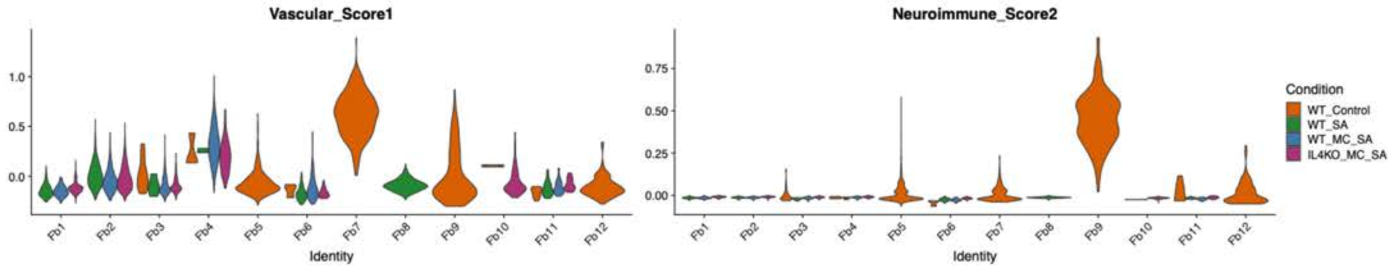

X

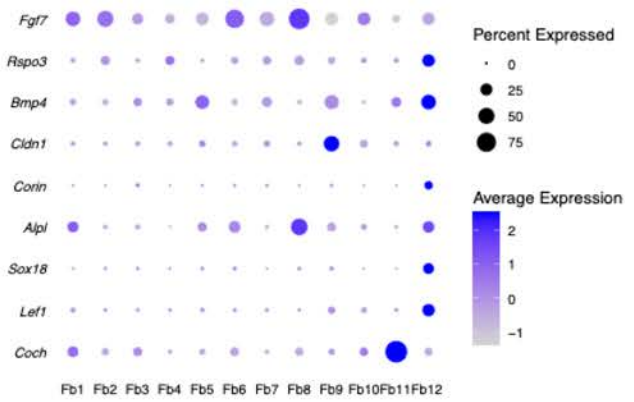

Y

Feature plot of *Il4ra* expression in Fibroblast clusters across conditions

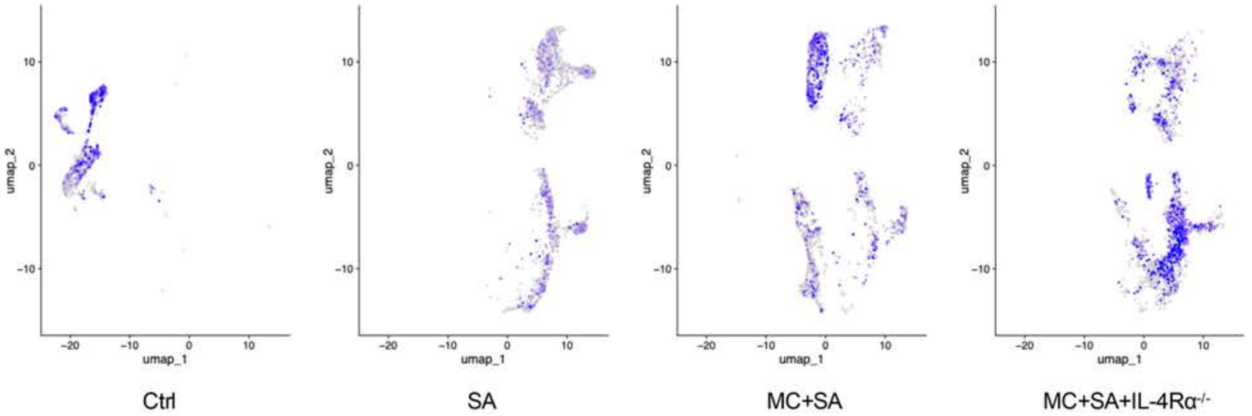

Figure S3

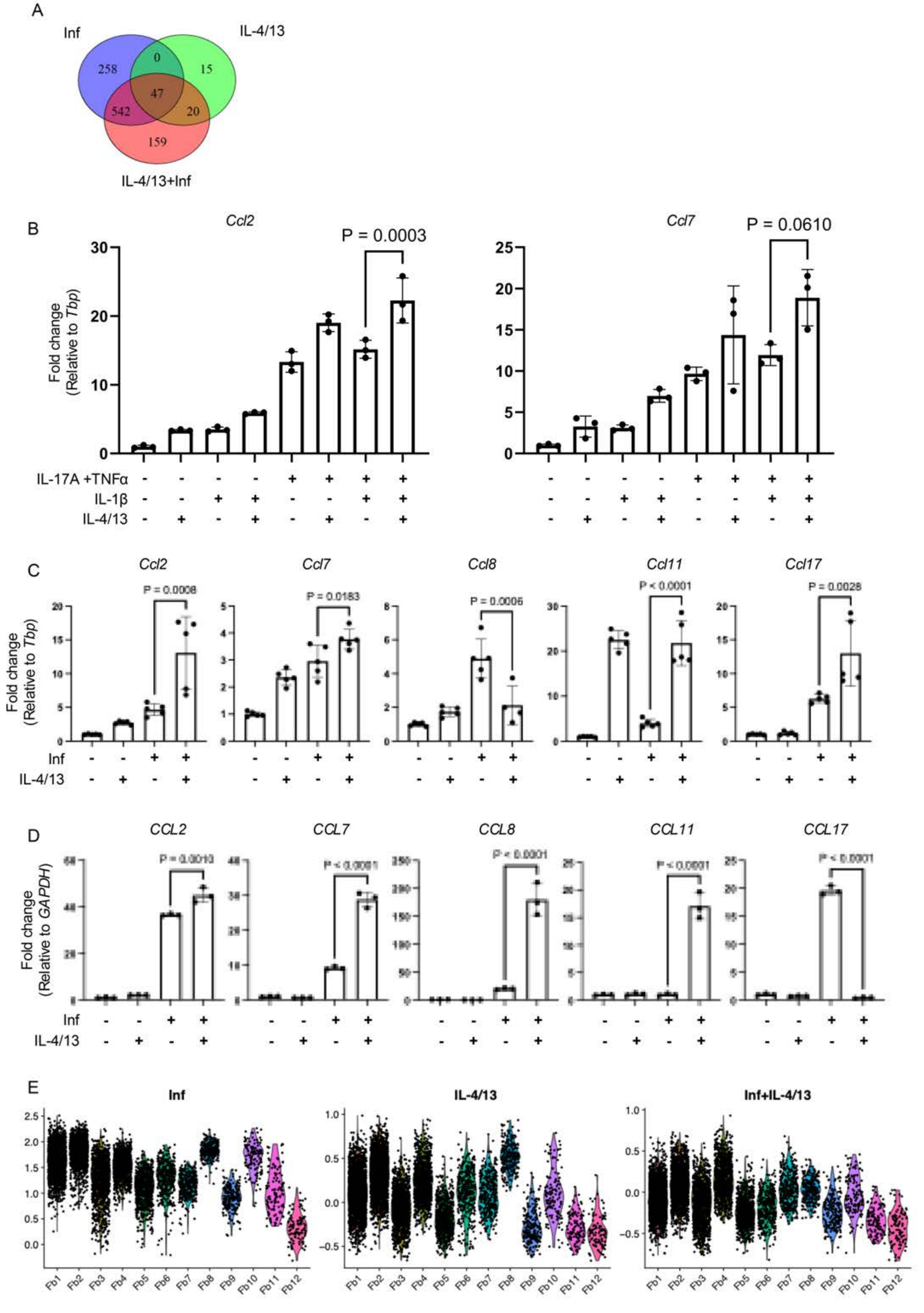

A
Inf
IL-4/13
IL-4/13+Inf
258
0
15
47
542
20
159
B
Ccl2
P = 0.0003
Fold change (Relative to Tbp)
Ccl7
P = 0.0610
IL-17A +TNFα
IL-1β
IL-4/13
C
Ccl2
P = 0.0008
Ccl7
P = 0.0183
Ccl8
P = 0.0006
Ccl11
P < 0.0001
Ccl17
P = 0.0028
Inf
IL-4/13
D
CCL2
CCL7
CCL8
CCL11
CCL17
Fold change (Relative to GAPDH)
E
Inf
IL-4/13
Inf+IL-4/13
Fb1
Fb2
Fb3
Fb4
Fb5
Fb6
Fb7
Fb8
Fb9
Fb10
Fb11
Fb12

Figure S4

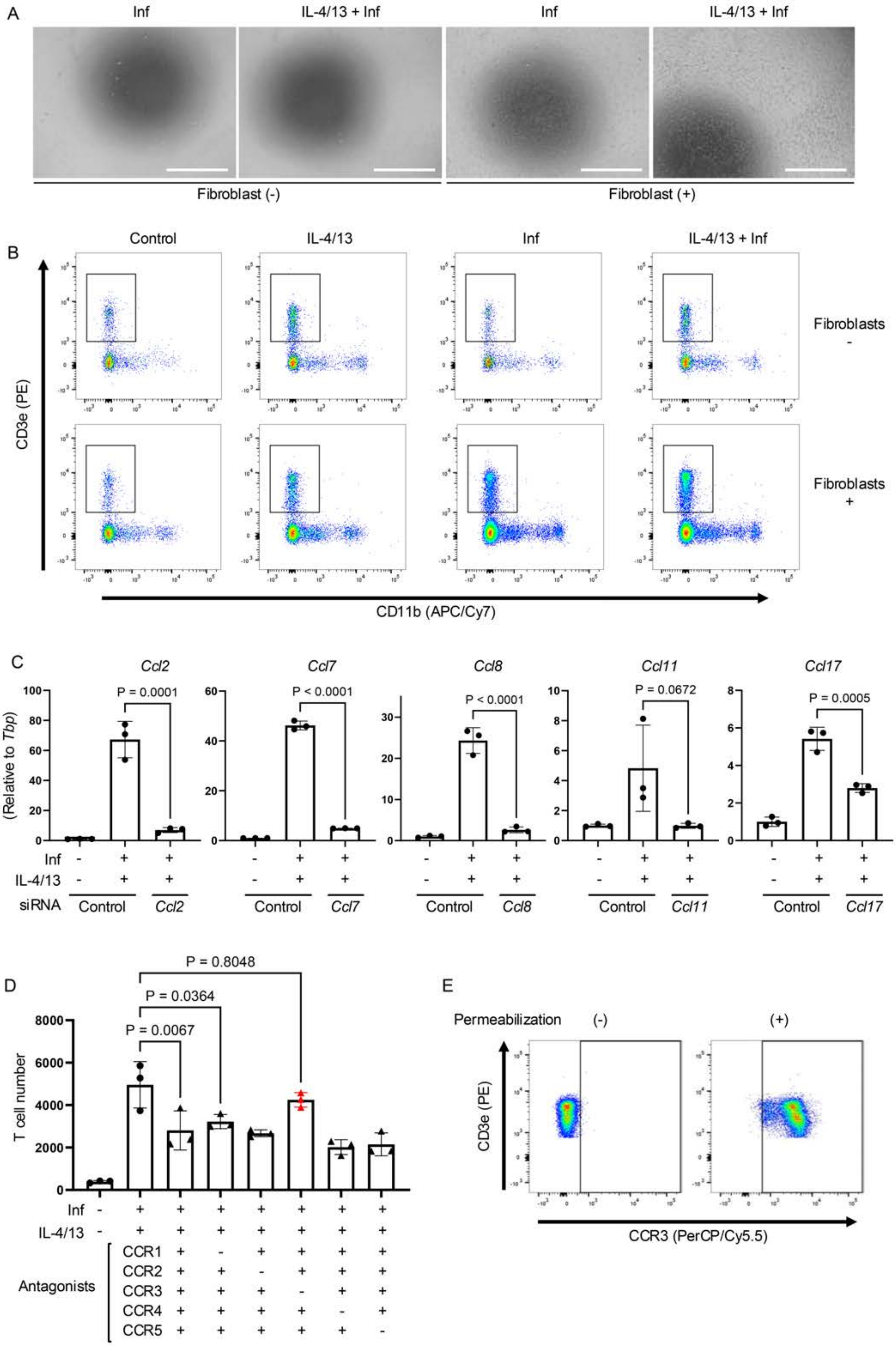

A
Inf
IL-4/13 + Inf
Inf
IL-4/13 + Inf
Fibroblast (-)
Fibroblast (+)
B
Control
IL-4/13
Inf
IL-4/13 + Inf
Fibroblasts -
Fibroblasts +
CD3e (PE)
CD11b (APC/Cy7)
C
Ccl2
Ccl7
Ccl8
Ccl11
Ccl17
P = 0.0001
P < 0.0001
P < 0.0001
P = 0.0672
P = 0.0005
Fold change (Relative to Tbp)
Inf
IL-4/13
siRNA
Control
Ccl2
Ccl7
Ccl8
Ccl11
Ccl17
D
P = 0.8048
P = 0.0364
P = 0.0067
T cell number
Inf
IL-4/13
Antagonists
CCR1
CCR2
CCR3
CCR4
CCR5
E
Permeabilization
(-)
(+)
CD3e (PE)
CCR3 (PerCP/Cy5.5)

Figure S5

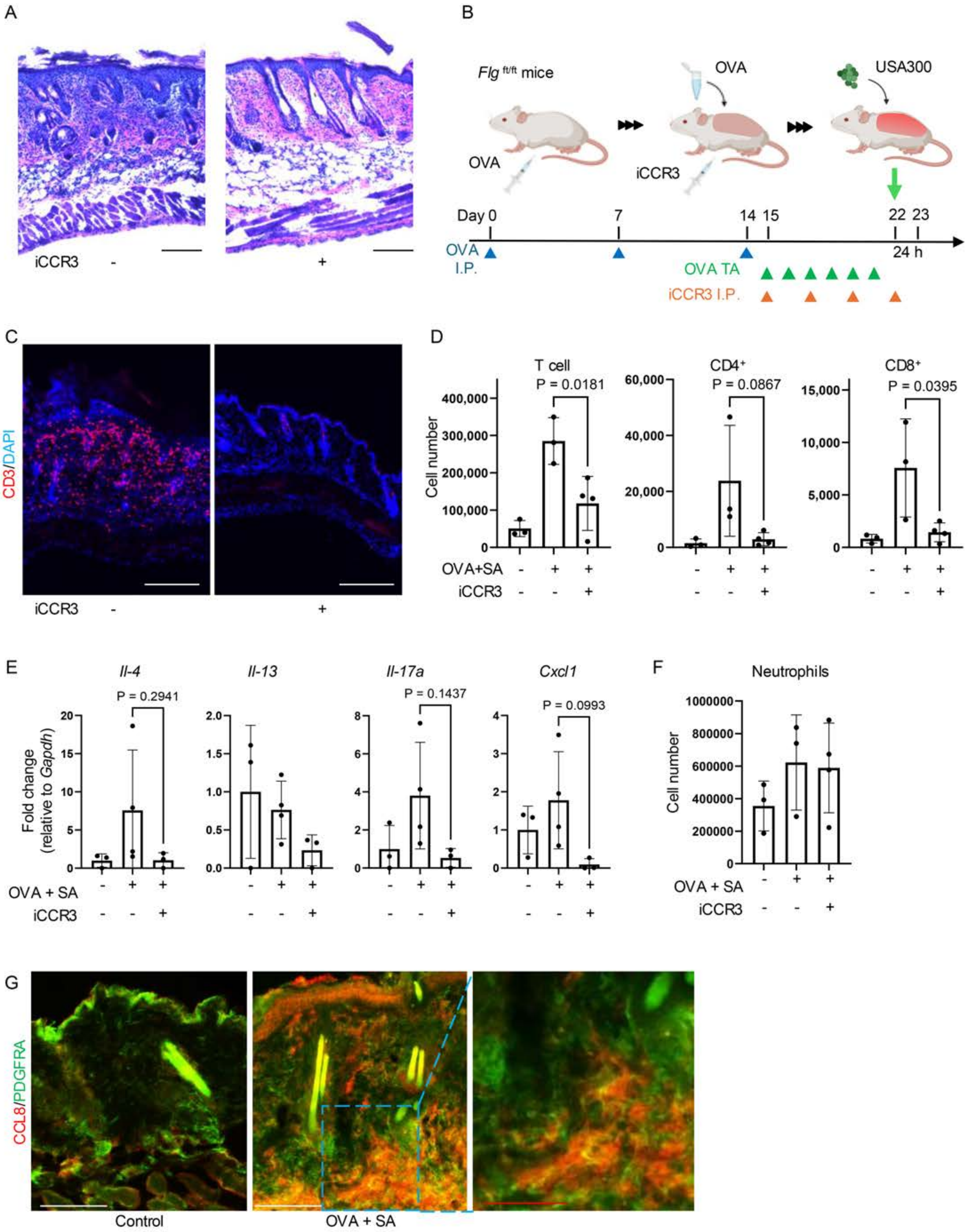
A
iCCR3 - +
B
Flg ft/ft mice
OVA
OVA
iCCR3
USA300
Day 0
7
14 15
22 23
24 h
OVA I.P.
OVA TA
iCCR3 I.P.
C
CD3/DAPI
iCCR3 - +
D
T cell
P = 0.0181
CD4+
P = 0.0867
CD8+
P = 0.0395
Cell number
OVA+SA - + +
iCCR3 - - +
E
Il-4
P = 0.2941
Il-13
Il-17a
P = 0.1437
Cxcl1
P = 0.0993
Fold change (relative to Gapdh)
OVA + SA - + +
iCCR3 - - +
F
Neutrophils
Cell number
OVA + SA - + +
iCCR3 - - +
G
CCL8/PDGFRA
Control
OVA + SA
